# Postnatal environment affects auditory development and sensorimotor gating in a rat model for autism spectrum disorder

**DOI:** 10.3389/fnins.2025.1565919

**Published:** 2025-03-11

**Authors:** Ella Elizabeth Doornaert, Alaa El-Cheikh Mohamad, Gurwinder Johal, Brian Leonard Allman, Dorit Möhrle, Susanne Schmid

**Affiliations:** ^1^Anatomy and Cell Biology, Schulich School of Medicine & Dentistry, University of Western Ontario, London, ON, Canada; ^2^Faculty of Veterinary Medicine, University of Calgary, Calgary, AB, Canada; ^3^Department of Psychology, University of Western Ontario, London, ON, Canada

**Keywords:** *CNTNAP2*, auditory processing, startle, prepulse inhibition, gap detection, rat, auditory brainstem response, neurodevelopmental disorders

## Abstract

The homozygous *Cntnap2* knockout (KO) rat is a well-established genetic model for neurodevelopmental disorders, exhibiting core features of autism spectrum disorder (ASD), including impaired sensory processing and sensorimotor gating. Recent findings indicate that the severity of ASD-like phenotypes in *Cntnap2* KO offspring is influenced by the parental genotype, with more pronounced impairments observed in KO rats bred from homozygous pairs compared to heterozygous pairs (*Cntnap2* HET). However, it is unclear to what extent this is due to *in utero* versus postnatal effects. We, therefore, investigated how early postnatal environmental factors, shaped by differences in parental and littermate genotypes, influence auditory processing and sensorimotor gating in *Cntnap2* KO rats. To examine this, we cross-fostered *Cntnap2* KO pups bred from *Cntnap2* KO rats to be reared with litters of *Cntnap2* HET dams. Cross-fostering *Cntnap2* KO rats reversed or partially reversed delayed hearing sensitivity maturation, heightened acoustic startle responses, and deficits in prepulse inhibition of the acoustic startle response. However, cross-fostering also exacerbated deficits in the neural responsiveness and conductivity in the auditory brainstem, as well as in gap-induced prepulse inhibition of the acoustic startle response. These results emphasize the importance of considering the postnatal environment and breeding strategies in preclinical genetic models of neuropsychiatric disorders. More importantly, they also demonstrate that ASD-like traits, including alterations in brainstem sensory processing, are not strictly determined by genetic factors, but remain malleable by environmental factors during early postnatal development.

## Introduction

Autism spectrum disorder (ASD) is a neurodevelopmental condition caused by genetic, environmental, or most often, a combination of both factors (Lord et al., 2018). One genetic risk factor of ASD are variants in the contactin-associated protein-like 2 (*CNTNAP2*) gene, and a complete *CNTNAP2* loss-of-function causes a syndromic disorder with core symptoms of autism (Strauss et al., 2006; Arking et al., 2008). *CNTNAP2* encodes for the neurexin CASPR2, a transmembrane protein highly expressed in sensory pathways during the critical period of brain development (Gordon et al., 2016). The homozygous *Cntnap2* knockout (KO) rat is a validated preclinical model of ASD that consistently exhibits ASD-like traits, including sensory processing alterations similar to the ones often observed in autistic individuals (Scott et al., 2018, 2020, 2022; Möhrle et al., 2021; El-Cheikh Mohamad et al., 2023; Doornaert et al., 2024). Auditory processing disruptions in ASD are at least partly due to delayed development of the auditory brainstem (Roth et al., 2012; Miron et al., 2016, 2018). Correspondingly, *Cntnap2* KO rats showed delayed maturation of the auditory brainstem response (ABR), resulting in lower and slower ABR waves (Scott et al., 2018). Like many autistic individuals, *Cntnap2* KO rats also exhibit heightened acoustic startle responses to sudden loud noises and reduced prepulse inhibition (PPI), the attenuation of the startle response when a less intense stimulus precedes the startle stimulus (Scott et al., 2018, 2020; Möhrle et al., 2021; El-Cheikh Mohamad et al., 2023; Doornaert et al., 2024).

“Critical periods” of brain development are time windows of increased plasticity in early postnatal development when neural circuits demonstrate a heightened sensitivity to environmental inputs (Inguaggiato et al., 2017). During critical periods, sensory deprivation can result in significant and often irreversible changes in neural circuitry (Kral et al., 2001; Chang and Merzenich, 2003; Bi et al., 2006; Bures et al., 2010; Bureš et al., 2017). Conversely, enriched sensory environments can enhance plasticity and promote optimal development of sensory pathways, highlighting the critical role of sensory experiences during early life (Engineer et al., 2004; Percaccio et al., 2007; Bureš et al., 2014; Svobodová Burianová and Syka, 2020). Indeed, evidence suggests that environmental factors during the critical period of auditory development can modulate phenotypic outcomes in *Cntnap2* KO rats. Möhrle et al. (2023) examined the maternal isolation-induced ultrasound vocalizations of *Cntnap2* KO pups bred from both heterozygous *Cntnap2* knockout (*Cntnap2* HET) and *Cntnap2* KO pairings. Whereas the ultrasound vocalizations of *Cntnap2* KO pups bred from heterozygous parents (KO^het^) were only slightly different from wildtype controls, *Cntnap2* KO pups from homozygous KO pairings (KO^hom^) exhibited a higher number of calls with different temporal structure characteristics, altered call pitch, and less consistency in transitioning between call types. Similarly, *in vitro* patch-clamp electrophysiology revealed differences in the intrinsic neuronal properties and synaptic activity in auditory cortex pyramidal neurons between *Cntnap2* KO^het^ and KO^hom^ rats (Scott et al., 2022; Mann et al., 2023). Lastly, delayed ABR maturation was observed in *Cntnap2* KO^hom^ rats but not in *Cntnap2* KO^het^ pups (Scott et al., 2018; Zheng et al., 2023).

In summary, evidence suggests that brain alterations caused by the *Cntnap2* mutation may depend on differences between heterozygous and homozygous breeding. These differences could emerge prenatally, due to the maternal *in utero* environment and/or maternal mitochondrial DNA, or postnatally, caused, e.g., by differences in maternal care or sibling social and communicative interactions. It also remains unclear to what extent the developmental consequences resulting from the *Cntnap2* mutation are still malleable and sensitive to early environmental conditions. To examine this, the current study used a cross-fostering paradigm: *Cntnap2* KO^hom^ pups were cross-fostered to be reared by a heterozygous dam in her litter prior to the critical period of auditory development (KO^CF^). The ABR, startle reactivity, and PPI were compared between *Cntnap2* KO^CF^ animals and non-cross-fostered KO^hom^ and KO^het^ animals. We hypothesized that cross-fostering benefits the development of neural circuits underlying auditory processing, potentially restoring hearing sensitivity maturation and reducing heightened acoustic startle response and PPI deficits.

## Materials and methods

### Experimental design

#### Animals

This study was conducted using male (M) and female (F) Sprague–Dawley wildtype rats (*Cntnap2* WT) and *Cntnap2* KO rats. A total of 14 litters were used in this study consisting of 9 litters from *Cntnap2* HET breeders (*Cntnap2* HET × *Cntnap2* HET) and 5 litters from *Cntnap2* KO breeders (*Cntnap2* KO × *Cntnap2* KO). Original *Cntnap2* HET breeders (*Cntnap2^em1Sage^*) were obtained from Horizon Discovery (Boyertown, PA, United States). *Cntnap2* KO breeders were obtained from in-house breeding of *Cntnap2* HET pairs. Rats were housed in open cages and given *ad libitum* food and water. Holding rooms were temperature-controlled and kept on a 12-h light/dark cycle. All behavioral testing occurred during this cycle’s light phase (from 7:00 to 19:00 h). All procedures were approved by the University of Western Ontario Animal Care Committee and followed the guidelines provided by the Canadian Council on Animal Care.

Five breeding rounds were initially conducted with at least 2 weeks in between. One breeding round involved pairing two *Cntnap2* HET males with two *Cntnap2* HET females each, as well as one *Cntnap2* KO male with two *Cntnap2* KO females. Pairings used males aged 13 weeks to 11 months and females aged 11 weeks to 11 months. Unrelated males and females (non-siblings) were used for pairings. All females that were impregnated were only used once as first-time dams. All males that impregnated a female were only used once, except for one *Cntnap2* KO male that impregnated two females from consecutive rounds.

**Figure 1:**
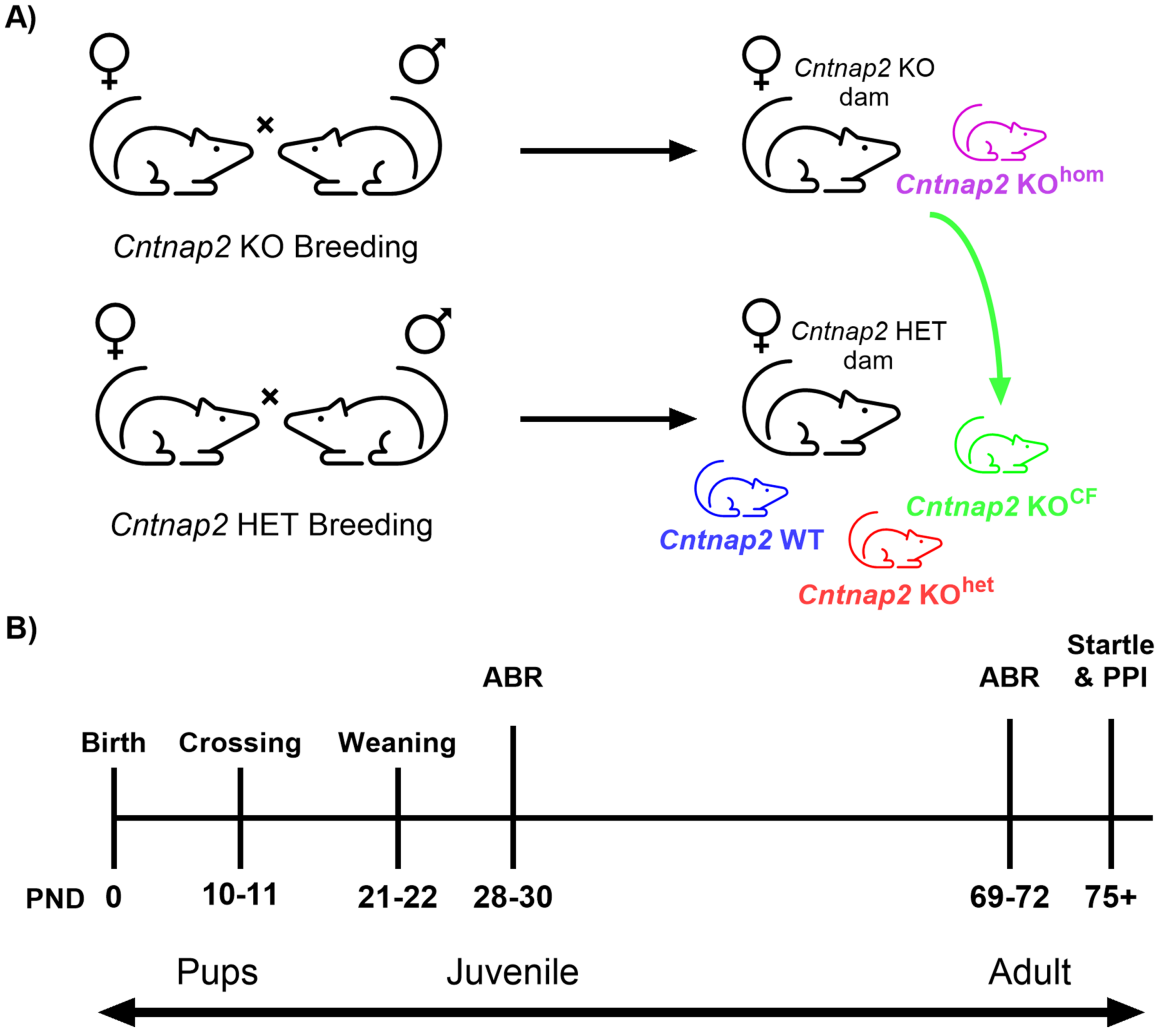
Cross-fostering illustration and summary of experimental timeline. **(A)** A breeding round consisted of breeding *Cntnap2* KO (*Cntnap2*- KO × *Cntnap2* KO) and *Cntnap2* HET (*Cntnap2* HET × *Cntnap2* HET) rats. Experimental groups included: *Cntnap2* WT animals bred by *Cntnap2* HET rats and reared by a *Cntnap2* HET dam (*Cntnap2* WT; blue), *Cntnap2* KO animals bred by *Cntnap2* HET rats and reared by a *Cntnap2* HET dam (*Cntnap2* KO^het^; red), *Cntnap2* KO animals bred by *Cntnap2* KO rats and reared by a *Cntnap2* KO dam (*Cntnap2* KO^hom^; purple), and *Cntnap2* KO animals bred by *Cntnap2* KO rats and cross-fostered to be reared by a *Cntnap2* HET dam (*Cntnap2* KO^CF^; green). **(B)** Experimental timeline outlining the postnatal days (PND) for cross-fostering and behavioral assessments. The auditory brainstem response (ABR) was assessed during the juvenile period and adulthood, while the acoustic startle response and prepulse inhibition (PPI) were evaluated in adulthood.

Rats were paired for 48 h to ensure that pregnant dams would have a similar date of birth within a breeding round. Females paired together for breeding were separated into single cages three to 4 days before their expected date of giving birth. After birth, toe clips were taken for offspring between postnatal (PND) 5 and 6 to genotype *Cntnap2*. Between PND 10 and 11, litters from *Cntnap2* HET pairings were culled by euthanizing all the *Cntnap2* HET pups so that only *Cntnap2* WT and *Cntnap2* KO (KO^het^) offspring remained (Figure 1A; *Cntnap2* WT animals are represented in blue, *Cntnap2* KO^het^ animals are represented in red). At that time, 2 to 5 *Cntnap2* KO pups from *Cntnap2* KO pairings were transferred to these culled litters of *Cntnap2* HET pairings (group *Cntnap2* KO^CF^; represented in green). The remainder of the *Cntnap2* KO offspring from the *Cntnap2* KO pairings remained with the *Cntnap2* KO dam for rearing (group *Cntnap2* KO^hom^; represented in purple). The cross-fostering time point of PND 10 to 11 was chosen balancing the time it took to receive the genotyping results with the start of the critical period of auditory development upon opening of the meatus at around PND 12 (Figure 1B). It is expected that the pups did not receive functional auditory input until this point (Bi et al., 2006).

To cross-foster the pups from the litter of a *Cntnap2* KO to a *Cntnap2* HET dam, both dams were removed from their home cages and a heating pad was placed under the cages to keep the pups warm. Before crossing, *Cntnap2* KO^CF^ pups were covered and rubbed in the nesting material of the *Cntnap2* HET dam for 3 min to make them smell like the pups of the *Cntnap2* HET dam. This process of rubbing the animals with bedding from their final cage was performed on all the animals to maintain consistency. Gloves were changed between the handling of animals of different litters to reduce the scents of foreign litters from carrying over. Then, dams were returned to their home cages and left undisturbed for 48 h (cages not changed). Across all breeding rounds, all cross-fostered pups were accepted by their foster dam. Animals were weaned between PND 21 and 22 and housed in cages of two to three sex-matched siblings from their final litter. When possible, *Cntnap2* KO^CF^ animals were housed with one or two each of *Cntnap2* WT and KO^het^ animals.

After five breeding rounds, an additional two rounds were conducted with only *Cntnap2* HET pairings to increase the sample size for the *Cntnap2* WT and KO^het^ animal groups. For these rounds, the procedure of cross-fostering animals into these litters was mimicked by introducing *Cntnap2* HET animals from other *Cntnap2* HET pairings.

#### Auditory brainstem response

ABR recordings were conducted during the juvenile stage and adulthood. 90 juvenile rats were tested at PND 28–30: 26 *Cntnap2* WT rats (13 male/13 female), 21 *Cntnap2* KO^het^ (11 male/10 female), 23 *Cntnap2* KO^hom^ (12 male/11 female), and 20 *Cntnap2* KO^CF^ (10 male/10 female). A total of 81 adult rats were tested at PND 69–72: 21 *Cntnap2* WT rats (11 male/10 female), 18 *Cntnap2* KO^het^ (10 male/ 8 female), 22 *Cntnap2* KO^hom^ (12 male/10 female), and 20 *Cntnap2* KO^CF^ (10 male/10 female). Rats were anesthetized with ketamine (juvenile: 40 mg/kg or adult: 80 mg/kg) and xylazine (juvenile: 2.5 mg/kg or adult: 5 mg/kg). For juveniles, dosing intervals were all IM. and administered as follows: initial full dose +1/4 dose, 1/4 dose after 5 min, 1/3 dose after 20–30 min, and 1/4 dose every 30 min after as necessary. For adults, the initial full dose +1/4 dose was administered IP. and supplemental doses were administered IM as follows: 1/3 dose after 15 min and 1/4 dose every 30 min as necessary. For both juveniles and adults, supplemental doses were switched to ketamine only after administering the supplemental doses totaling the initial full dose. Injections of 0.9% saline were provided every hour (10 mL/kg) and eye lubrication was applied before testing. Throughout the recordings, body temperature was maintained at approximately 37°C using a homeothermic heating pad (ATC-2000; World Precision Instruments).

Subdermal electrodes (27 gauge; Rochester) were positioned over the right mastoid process (active electrode), the vertex (reference electrode), and the midback (ground electrode). The acoustic stimuli used in the ABR assessment consisted of a click and 4 tones (4 kHz, 11 kHz, 20 kHz, and 32 kHz; 10 ms duration; 1 ms rise/fall time), which were generated using a Tucker-Davis Technologies RZ6 processing module and sampled at 100 kHz. A magnetic speaker (MF1; Tucker-Davis Technologies) positioned 10 cm from the animal’s right ear was used to deliver the stimuli and its left ear was blocked with a custom foam plug. Before the ABR assessment, the acoustic stimuli were calibrated with custom MATLAB software (The Math-Works) using a 1/4-inch microphone (2,530; Larson Davis) and preamplifier (2,221; Larson Davis). The acoustic stimuli were each presented 750 times (21 times/s) at decreasing intensities from 90 to 0 dB sound pressure level (SPL) in 5 dB SPL steps. The sound-evoked activity associated with the ABR assessment was preamplified and digitized using a Medusa4Z BioAmp (Tucker-Davis Technologies) set to a 12 kHz sampling frequency. A fiber-optic cable sent the digitized signal to the RZ6 processing module. The signal was filtered (300–3,000 Hz) and averaged using BioSig software (Tucker-Davis Technologies). The positive and negative peak amplitudes of each of the characteristic waves of the rat ABR were measured in microvolts in reference to the baseline (0 V) and the latency of each of these peaks was determined from the stimulus onset.

**Figure 2:**
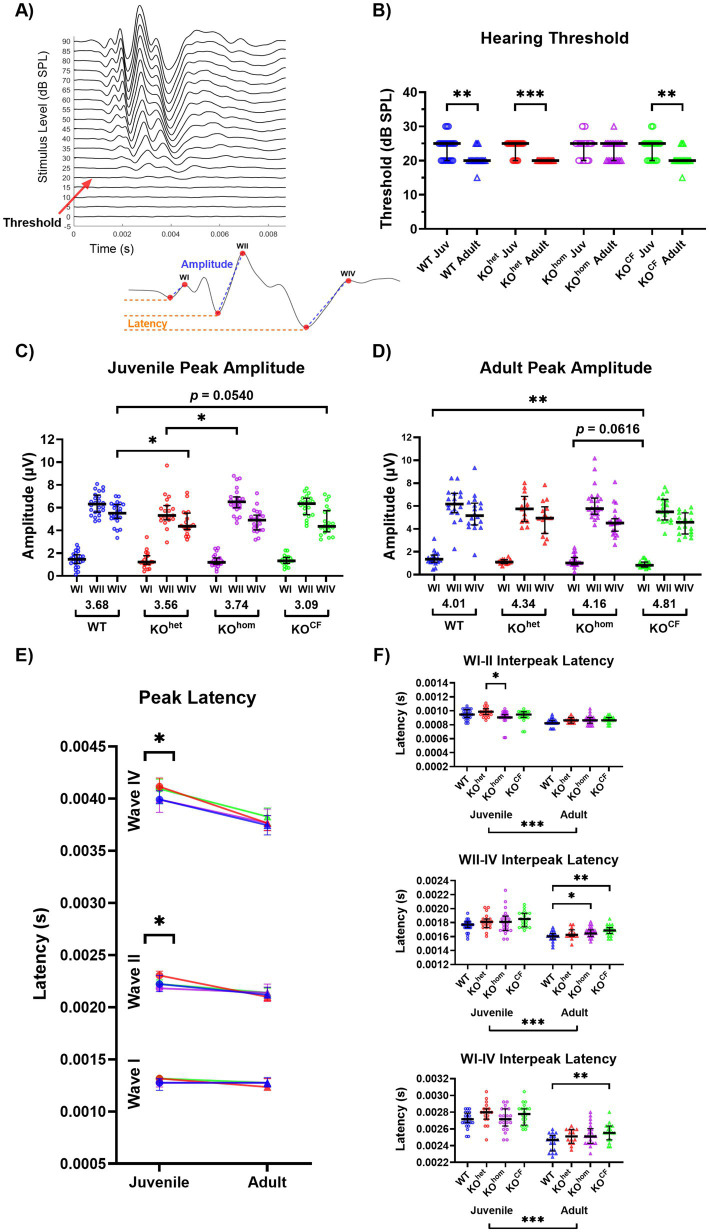
Cross-fostered *Cntnap2* knockout rats have improved development of hearing sensitivity but reduced neural responsiveness and conduction within the auditory brainstem pathway. **(A)** An example of the acoustically evoked ABRs from a rat in response to click stimuli of increasing sound level with a red arrow to indicate the ABR threshold. Below is a magnification of an ABR trace from the same animal in response to a 70 dB SPL click stimulus. Waves I, II, and IV reflect the synchronized neural activity in the auditory nerve, cochlear nucleus, and inferior colliculus/lateral lemniscus, respectively. Measurements show how amplitude (from negative to positive peak; blue) and peak latency (negative peak; orange) were determined. **(B)** Change in ABR threshold to click stimuli with age. The hearing threshold of *Cntnap2* KO^hom^ rats does not improve between juvenile age and adulthood as observed in WT and KO^het^ rats. *Cntnap2* KO^CF^ rats exhibit typical maturation of hearing threshold with age, suggesting they have restored development of hearing sensitivity. **(C)** Juvenile ABR peak amplitudes. Group WIV/I ratios are indicated under the *x*-axis. Juvenile *Cntnap2* KO^hom^ and KO^het^ rats exhibit reduced peak amplitudes for WII and WIV, respectively, compared to WT rats. The WIV amplitude of *Cntnap2* KO^CF^ rats is trending toward being lower than WT rats. **(D)** Adult ABR peak amplitudes. Group WIV/I ratios are indicated under the *x*-axis. The reduced peak amplitudes observed in juvenile *Cntnap2* KO^hom^ and KO^het^ rats do not persist in adulthood. In contrast, *Cntnap2* KO^CF^ rats display reduced WI amplitude compared to WT rats. **(E)** ABR peak latencies across age. Only juvenile *Cntnap2* KO^het^ rats show slower WII and WIV latencies. This is normalized by adulthood. **(F)** ABR interpeak latencies. In adulthood, *Cntnap2* KO^hom^ and KO^CF^ exhibit slower WII-IV interpeak latencies. Adult *Cntnap2* KO^CF^ rats also show a slower WI-IV interpeak latency, indicative of overall slower conduction of the ABR. *p < 0.05, **p < 0.01, ***p < 0.0001.

The whole trace data were exported to MATLAB R2022a to determine the hearing sensitivity (ABR threshold) for the 5 sound stimuli as well as extract the values for the amplitudes and latencies for waves I, II, and IV resulting from the click stimuli (Figure 2A). Consistent with previous studies, each rat’s ABR threshold for the click and tonal stimuli was determined using the criterion of just noticeable deflection of the averaged electrical activity within a 10 ms window following the click (Schormans et al., 2017; Scott et al., 2018). Wave amplitudes were calculated by subtracting the negative peak from the positive peak amplitude (peak-to-peak). Central compensation of neuronal responsiveness with age was calculated by the ratio of ABR wave IV to I amplitudes (Möhrle et al., 2016). ABR peak latencies and interpeak latencies were based on the negative peak amplitudes. To normalize the amplitude and latency measures across animals, these measures were compared at sound levels above the animals’ thresholds (20 dB to 60 dB above threshold). The experimenter was blinded to the animal’s genotype for all analyses associated with the ABR assessment.

#### Acoustic startle response, PPI, gap-PPI

The acoustic startle response, PPI, and gap-induced prepulse inhibition of startle (gap-PPI) were evaluated in adulthood (PND 75+). A total of 81 rats were tested, including 21 *Cntnap2* WT rats (11 male/10 female), 19 *Cntnap2* KO^het^ (10 male/9 female), 21 *Cntnap2* KO^hom^ (11 male/10 female), and 20 *Cntnap2* KO^CF^ (10 male/10 female), using the Med Associates (Vermont, United States) startle system with protocols modified from Doornaert et al. (2024), El-Cheikh Mohamad et al. (2023) and Miller et al. (2021). In brief, animals were placed in plexiglass tubes on weight-transducing platforms in sound-attenuating startle boxes (Med Associates). Animals were initially acclimated to the experimental procedure by undergoing three 5-min sessions in the startle box with only background noise (65 dB sound pressure level, SPL, white noise). Then, a session to determine the startle reactivity (I/O function) was conducted consisting of 12 startle stimuli ranging from 65 dB to 120 dB in 5 dB increments (20 ms white noise) plus the two prepulse intensities used in the PPI protocol, 75 dB and 85 dB (4 ms white noise). Stimuli were presented in pseudorandomized order. This was done before testing to adjust the gain of the platform transducer signal to ensure optimal spread over the dynamic range of the system for each animal.

Testing of the acoustic startle response and PPI involved two sessions a day for five consecutive days. Each testing session consisted of three distinct blocks: acclimation, habituation, and PPI. The first block was 5 min of acclimation to the startle box with only background noise (65 dB white noise). The habituation block presented animals with 12 trials of a 110 dB startle stimulus (20 ms white noise; 10–15 s variable intertrial intervals). The data were only collected from the third block, in which PPI was assessed by pairing a non-startling prepulse (75 dB or 85 dB; 4 ms white noise) and a startle stimulus (70 dB, 80 dB, 90 dB, 100 dB, 110 dB, or 120 dB; 20 ms white noise) with a 100 ms fixed interstimulus interval. Startle-alone stimuli without prepulses were also presented. Trials had variable intertrial intervals of 10–15 s and were presented in a pseudorandomized order. Each condition was repeated 4 times per testing session, resulting in 84 trials per testing session and 40 repetitions per trial type across all 10 testing sessions.

Following PPI testing and a day of break without testing, gap-PPI testing was conducted over 3 consecutive days. The first day consisted of a 5-min acclimation session to the new background noise used for the gap-PPI protocol (75 dB, white noise). Then, a session to assess gap-PPI was done on days 2 and 3. These sessions paired gap periods (2 ms, 5 ms, 10 ms, 20 ms, 40 ms, 50 ms, 75 ms, or 100 ms) and a 115 dB startle stimulus (20 ms white noise; 100 ms fixed interstimulus interval). A startle-alone stimulus was also presented. Again, trials had variable intertrial intervals of 10–15 s and were presented in a pseudorandomized order. Each condition was repeated 10 times per testing session, resulting in 90 trials per testing session and 20 sessions total per trial type combined over the two sessions.

The startle magnitude was defined as the maximum peak-to-peak value of the response waveform. Before statistical analyses, each rat’s startle response values were corrected for the gain setting. The subsequent analysis followed the methods outlined by Martin-Iverson and Stevenson (2005) and Miller et al. (2021). Startle reactivity across the range of stimuli was assessed by fitting each animal’s responses to a sigmoidal regression function using GraphPad Prism 9.3.1 (San Diego, California, United States; Non-linear regression; Method: Sigmoidal, 4PL, X is concentration; Method: Least squares regression; Initial values: choose automatically; Confidence: Unstable parameters and ambiguous fits as Neither option; Diagnostics: default values including Adjusted R Squared, RMSE, and tests of normality, see also Möhrle et al., 2021) with the following equation:


$$Y=Bottom+X^{Hillslope}\left(\frac{Top-Bottom}{X^{Hillslope}+ES50^{Hillslope}}\right)$$

where *Y* represents the startle response magnitude, *Top* is the maximum startle response magnitude and *Bottom* is the minimum response magnitude. *X* denotes the startle stimulus intensity (dB SPL) necessary to elicit a certain Y value (in arbitrary units). *ES50* is the sound intensity (dB SPL) required to maintain the half-maximum response. *Hillslope* is the slope of the curve. Parameters of interest were derived from the equation to evaluate and compare differences in baseline startle and PPI. This includes the maximum startle response (*Top*), startle threshold (10% of maximum threshold), ES50, and saturation point (90% of maximum startle). In this sigmoidal regression analysis, GraphPad Prism provided the standard error of regression using Sy.x, which serves as an estimate of the goodness-of-fit for models involving two or more parameters.

Startle scaling was assessed through changes to the maximum startle response, or *Top* (El-Cheikh Mohamad et al., 2023; Doornaert et al., 2024). Each animal’s responses were fitted to a sigmoidal regression function as previously described (including Constrain: *Bottom* is constant equal to 0). Sound scaling was determined by changes to the threshold, ES50, and saturation point (Martin-Iverson and Stevenson, 2005; Möhrle et al., 2021; El-Cheikh Mohamad et al., 2023; Doornaert et al., 2024). For this, startle responses for each animal and prepulse condition were scaled between 0 and 1. To scale the startle magnitudes at each startle stimulus intensity (*X*), we used the following equation: (startle magnitude at *X—*startle magnitude at 70 dB startle stimulus) / (startle magnitude at 120 dB startle stimulus—startle magnitude at 70 dB startle stimulus). The scaled values were then fitted to the sigmoidal regression function using the same procedure as above (except Constrain: *Bottom* is constant equal to 0 and *Top* is constant equal to 1). ES50 was provided by the regression, as well as *Hillslope*, which can be used as a metric of reflex efficiency (Martin-Iverson and Stevenson, 2005). The threshold and saturation point were calculated in MATLAB R2022a by rearranging the above equation to solve for *X* (below). The threshold *Y* value was set to 10% of the *Top*, and the saturation point *Y* value was set to 90% of the *Top*.


$$X=\sqrt[{Hillslope}]{\frac{\left(Y-Bottom\right)\times \left(ES50^{Hillslope}\right)}{Top-Y}}$$

For PPI analysis, the percent PPI was calculated using the startle magnitudes from the 10 PPI sessions:


$$\%PPI=\left(1-\frac{startlemagnitudewithprepulse}{baselinestartlemagnitude}\right)\times 100\%$$

Similarly, the percent gap-PPI was obtained using the startle magnitude from the 2 gap-PPI sessions:


$$\%Gap-PPI=\left(1-\frac{startlemagnitudewith\;gap}{baselinestartlemagnitude}\right)\times 100$$

### Statistical analysis

The data are presented as group medians with errors indicating interquartile range (IQR). Outlier analysis was performed in IBM SPSS (version 26) for each testing component in this study: ABR, startle/PPI, and gap-PPI. Through boxplot assessment, extreme outliers were identified as those exceeding 3 IQR from the group median. Table 1 shows the number of animals per group for each behavioral test after outlier exclusion. Two additional *Cntnap2* KO^het^ females were excluded from the adult ABR analyses because their trace data were too noisy and illegible to extract reliable measures.

**Table 1:** The number of animals per group for each behavioral experiment after outlier exclusion.

		*Cntnap2* WT		*Cntnap2* KO^het^		*Cntnap2* KO^hom^		*Cntnap2* KO^CF^	
		Males	Females	Males	Females	Males	Females	Males	Females
ABR	Juvenile	12	12	11	8	12	11	10	10
	Adult	8	10	8	6	12	10	9	10
Startle & PPI	Adult	7	10	7	8	9	10	8	9
Gap-PPI	Adult	11	8	10	8	9	9	9	10

Subsequent statistical analyses were performed in GraphPad Prism 9.3.1 and RStudio 2022.07.2, and figures were generated in GraphPad Prism 9.3.1. To determine the main effects and interactions, we employed ARTool (Aligned Rank Transform, ART) to align-and-rank data for nonparametric factorial ANOVA, and ART-C for *post-hoc* pairwise comparisons (Wobbrock et al., 2011; Elkin et al., 2021). Statistical tests following the ART were based on the experimental design and included univariant analysis of variance [x-way ANOVA, repeated measures (RM) ANOVA, or Mixed-effects model, as appropriate], followed by multiple comparison tests with correction for type 1 error after Tukey’s method or Sidak’s multiple comparison test when appropriate. For measures in which there was no effect of sex or an interaction effect involving sex, the data were collapsed across sex to examine the effect of genotype and/or age. The chosen statistical significance level was *α* = 0.05. Resulting *p* values are reported in the figure captions using: no asterisk or ns for non-significance, *p < 0.05; **p < 0.01; ***p < 0.0001.

## Results

### Cross-fostering improves the maturation of hearing sensitivity in *Cntnap2* KO rats but exacerbates ABR responsiveness and latency impairments

We compared ABR thresholds to click stimuli from juvenile age and adulthood to assess the development of hearing sensitivity (Figures 2A,B). We found a significant effect of age on the threshold [*p* < 0.0001, *F*(1, 63) = 43.41]. Considering there was no effect of sex, nor interaction effects involving sex, the data across sex were collapsed for further analysis. After collapsing across sex, the effect of age on the threshold persisted, in addition to the appearance of an interaction effect between genotype and age [age *p* < 0.0001, *F*(1, 67) = 18.99; genotype × age *p* = 0.0011, *F*(3, 67) = 2.866]. *Post-hoc* testing revealed disrupted maturation of hearing sensitivity in *Cntnap2* KO^hom^ rats: whereas *Cntnap2* WT and KO^het^ rats exhibited a decrease in their ABR thresholds between the juvenile and adult stages, thresholds did not decrease in *Cntnap2* KO^hom^ [*Cntnap2* WT *p* = 0.0067, *Cntnap2* KO^het^ < 0.0001, *Cntnap2* KO^hom^ *p* = 0.6323; Figure 2B]. Interestingly, *Cntnap2* KO^CF^ rats displayed a decrease in threshold with age (*p* = 0.0011), indicating restored maturation of the brainstem upon cross-fostering. Please note that this finding was only observed for the threshold of click stimuli, whereas there were only subtle group differences for the threshold for tone stimuli (see Supplementary Figure 1).

Next, we examined the peak-to-peak amplitude and negative peak latency of WI, WII, and WIV of the ABR, representing sound processing in the auditory nerve, cochlear nucleus, and inferior colliculus/lateral lemniscus, respectively (Figures 2A,C–E). Moreover, we examined the interpeak latencies between WI-II, WII-IV, and WI-IV (Figure 2F). The analysis was first conducted across sound intensities 20 dB to 60 dB above the threshold and is shown in Supplementary Figures 2–4. This analysis revealed subtle group differences for all measures, and the sound level substantially interacted with all other factors in the analysis. Consequently, we chose to examine these measurements at the sound level 60 dB above the threshold, as this is where group differences appeared to emerge but were undetected by our first analysis. Again, the data were collapsed across sex for further analysis because there were no effects of sex nor interaction effects involving sex on these measures.

During the juvenile stage, there was a main effect of genotype on the amplitude of WII and WIV in response to a 60 dB click [WII *p* < 0.0001, *F*(1, 67) = 18.9; WIV *p* < 0.0001, *F*(1, 67) = 18.9; Figure 2C]. *Post-hoc* testing revealed that *Cntnap2* KO^het^ animals had a lower WII amplitude than KO^hom^ animals (*p* = 0.0323). *Cntnap2* KO^het^ animals also had a lower WIV amplitude than WT animals, and KO^CF^ animals showed a similar trend (*Cntnap2* KO^het^ *p* = 0.0418, *Cntnap2* KO^CF^ *p* = 0.0540). In adulthood, an effect of genotype on amplitude was only found in WI [*p* = 0.0036, *F*(3, 69) = 4.959; Figure 2D], where *Cntnap2* KO^CF^ animals had lower amplitudes than WT animals and were trending toward having lower amplitude than *Cntnap2* KO^hom^ (*Cntnap2* WT *p* = 0.0018, *Cntnap2* KO^hom^ *p* = 0.0616).

Based on these age-related findings, we calculated the change in amplitude with age for each wave by dividing each animal’s juvenile response by the adult response and taking its percentage. A significant effect of genotype was found only in WI [*p* = 0.038, *F*(3, 67) = 2.96; Supplementary Figure 6A], where *Cntnap2* KO^CF^ animals had a greater change in amplitude than WT animals (*p* = 0.0434).

We also calculated the WIV/I ratio, a measure reflecting the maturation of neural processing in the auditory brainstem (Scott et al., 2018; Möhrle et al., 2019). A higher ratio typically indicates a more mature auditory system, while a lower ratio suggests immaturity or delayed development. Across all the sound levels above threshold, only subtle group differences were found, except that *Cntnap2* KO^CF^ females showed greater WIV/I ratios in adulthood compared to juvenile age at the higher sound intensities (Supplementary Figure 5). At 60 dB above the threshold, there was no effect of genotype within the juvenile or adult stage for the WIV/I ratio [juvenile *p* = 0.6968, *F*(3, 82) = 0.4803; adult *p* = 0.1525, *F*(3, 69) = 1.815; group ratios are indicated under the *x*-axes in Figures 2C,D]. We calculated the percent change in WIV/I between juvenile age and adulthood and found no significant effect of genotype [*p* = 0.1001, *F*(3, 67) = 2.16; Supplementary Figure 6B].

We observed a significant effect of genotype on peak latencies in the juvenile age for WII and WIV [WII *p* = 0.0157, *F*(3, 82) = 3.662; WIV *p* = 0.0116, *F*(3, 82) = 3.907; Figure 2E]. *Post-hoc* testing revealed that *Cntnap2* KO^het^ animals had a greater WII latency than KO^hom^ animals and a greater WIV latency than WT animals (WII *p* = 0.0057, WIV *p* = 0.0393). By adulthood, this slower latency appeared to normalize, as no significant genotype effects were found for any of the waves [WI *p* = 0.3311, *F*(3, 69) = 1.161; WII *p* = 0.8178, *F*(3, 69) = 0.3103; WIV *p* = 0.1541, *F*(3, 69) = 1.806].

When examining interpeak latencies, there was a significant effect of genotype for WI-II in juvenile animals [*p* = 0.0458, *F*(3, 82) = 2.788], where *Cntnap2* KO^hom^ animals showed a lower WI-II interpeak latency than KO^het^ animals (*p* = 0.0306; Figure 2F). In adulthood, there was an effect of genotype on WII-IV and WI-IV interpeak latencies [WII-IV *p* = 0.0063, *F*(3, 69) = 4.466; WI-IV *p* = 0.0147, *F*(3, 69) = 3.755]. Interestingly, both adult *Cntnap2* KO^hom^ and KO^CF^ animals had greater WII-IV interpeak latency than WT animals (*Cntnap2* KO^hom^ *p* = 0.0459, *Cntnap2* KO^CF^ *p* = 0.0042), and only KO^CF^ animals had a greater WI-IV interpeak latency than WT animals (*Cntnap2* KO^hom^ *p* = 0.1178, *Cntnap2* KO^CF^ *p* = 0.0045). We calculated the percent change in interpeak latency with age and found a significant effect of genotype for WI-II [*p* = 0.044, *F*(3, 67) = 2.815; Supplementary Figure 6C]. *Cntnap2* KO^hom^ animals had a lower change in WI-II interpeak latency with age compared to WT animals (*p* = 0.0388).

In summary, we found cross-fostering to have beneficial effects on the impaired maturation of hearing sensitivity as it restored the age-related decrease of hearing threshold in *Cntnap2* KO^CF^ rats upon reaching adulthood. However, cross-fostering did not benefit the changes in neural responsiveness or conductivity measured by the ABR. Rather on the contrary, cross-fostered animals appeared to have greater ABR disruptions as evidenced by a reduction in WI amplitude and a slower WI-IV interpeak latency.

### Cross-fostering reduces heightened startle response in *Cntnap2* KO males

**Figure 3:**
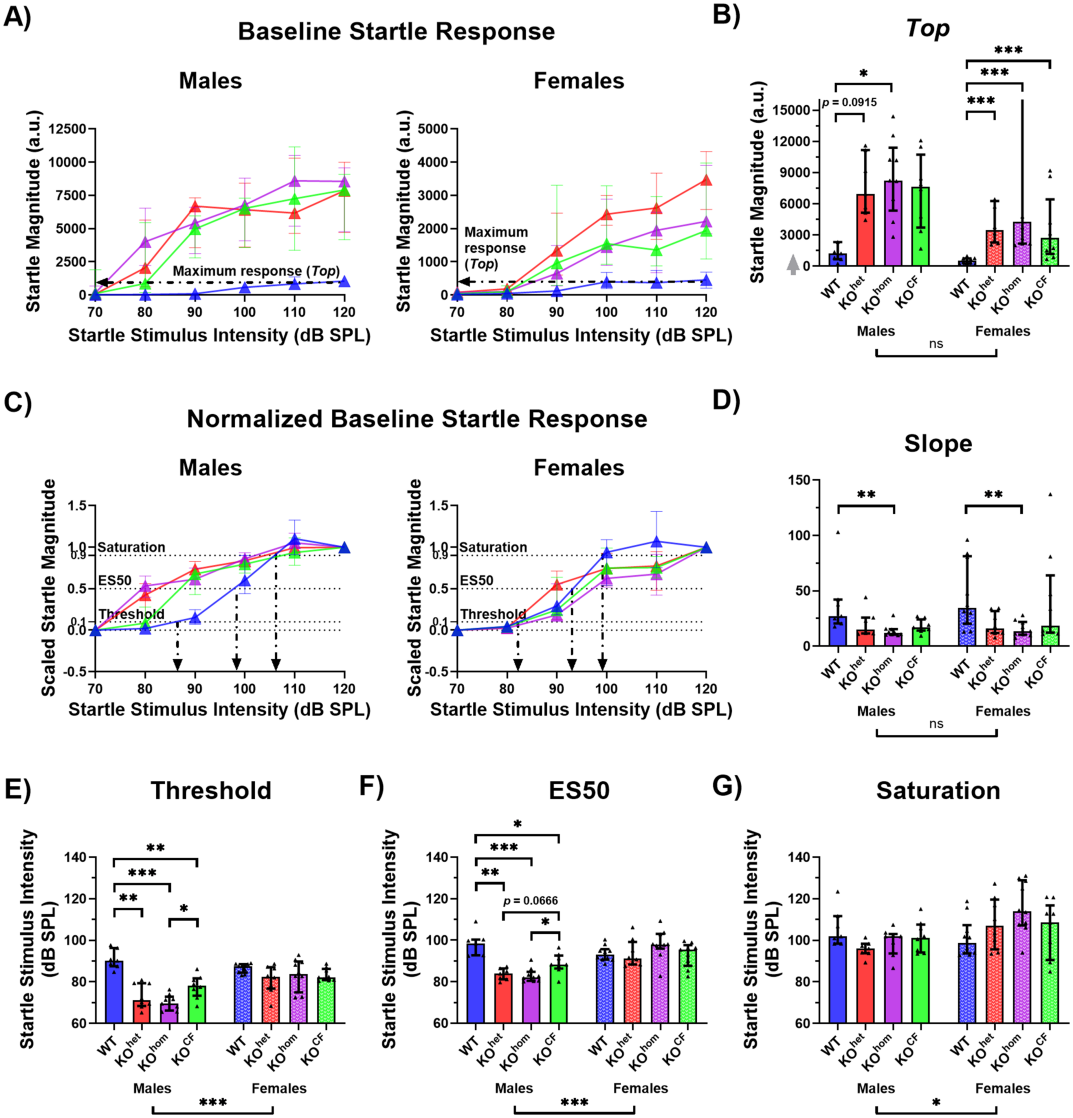
Cross-fostered *Cntnap2* knockout males show less exaggerated startle responses. **(A)** Baseline startle response curves. Black arrows point to the maximum startle response value (*Top*) for WT animals. Goodness of fit Sy.x: male WT = 449.7, male KO^het^ = 2,485, male KO^hom^ = 2,413, male KO^CF^ = 2,736, female WT = 181.1, female KO^het^ = 841.6, female KO^hom^ = 932.0, female KO^CF^ = 2,223. **(B)** Maximum startle response (*Top*). The gray arrow indicates that there are values outside the limits of the y-axis, but the graph was zoomed in to visualize the data more clearly. Unlike male *Cntnap2* KO^hom^ rats, KO^CF^ males do not have a statistically higher *Top* than WT males. This is not apparent in females where all *Cntnap2* KO rats have a higher *Top* than WT rats. **(C)** Scaled startle response curves. Black arrows point to the threshold, ES50, and saturation point for WT animals. Goodness of fit Sy.x: male WT = 0.2048, male KO^het^ = 0.1263, male KO^hom^ = 0.1311, male KO^CF^ = 0.1673, female WT = 0.1956, female KO^het^ = 0.1961, female KO^hom^ = 0.1823, female KO^CF^ = 0.2003. **(D)** Slope. In males and females, *Cntnap2* KO^hom^ rats but not KO^CF^ rats have a lower slope than WT rats. **(E)** Threshold. Male *Cntnap2* KO^CF^ rats have a higher threshold than KO^hom^ rats, but still a lower threshold than WT rats. **(F)** ES50. Male *Cntnap2* KO^CF^ rats have a higher ES50 than KO^hom^ rats, but still a lower ES50 than WT rats. **(G)** Saturation. There are no group differences in saturation point. *p < 0.05, **p < 0.01, ***p < 0.0001, ns indicates non-significance of the comparison.

After establishing a restoration of the ABR threshold upon maturation in cross-fostered animals and otherwise rather subtle changes in the ABR, we proceeded to test the effect of cross-fostering on baseline startle magnitude using the extracted parameters of the baseline startle I/O curves: maximum startle response (*Top*; Figures 3A,B), threshold, ES50, saturation point, and slope (Figures 3C–G). There were significant effects of sex for the threshold, ES50, and saturation [threshold *p* < 0.0001, *F*(1, 60) = 23.65; ES50 *p* < 0.0001, *F*(1, 60) = 31.61; saturation *p* = 0.0176, *F*(1, 60) = 5.959], as well as interaction effects between sex and genotype for the *Top*, threshold, ES50, and saturation [*Top p* = 0.0290, *F*(3, 60) = 3.218; threshold *p* = 0.0001, *F*(3, 60) = 8.004; ES50 *p* < 0.0001, ‑*F*(3, 60) = 11.63; saturation *p* = 0.0115, *F*(3, 60) = 4.007]. Therefore, for all parameters, the data were analyzed separately for males and females to assess the effect of genotype within the sexes.

In terms of maximum startle response, there was a significant effect of genotype for males and females [males *p* = 0.0337, *F*(3, 27) = 3.348; females *p* < 0.0001, *F*(3, 33) = 16.29]. Male *Cntnap2* KO^hom^ rats showed a higher *Top* value than WT rats (*p* = 0.0303), but *Cntnap2* KO^CF^ did not differ from WT rats (*p* = 0.13339; Figures 3A,B). All female *Cntnap2* KO rats showed a higher *Top* value than WT rats (*Cntnap2* KO^het^ *p* < 0.0001, *Cntnap2* KO^hom^ *p* < 0.0001, *Cntnap2* KO^CF^ *p* = 0.0009).

There was also a significant effect of genotype on startle thresholds in male, but not female rats [males *p* < 0.0001, *F*(3, 27) = 16.96; females *p* = 0.1196, *F*(3, 33) = 2.096]. All male *Cntnap2* KO groups showed a lower threshold than WT males (*Cntnap2* KO^het^ *p* = 0.0001, *Cntnap2* KO^hom^ *p* < 0.0001, *Cntnap2* KO^CF^ *p* = 0.0075), however, thresholds were less reduced in male *Cntnap2* KO^CF^ than in *Cntnap2* KO^hom^ males, as they significantly differed (*p* = 0.0136; Figures 3C,E). Similarly, there was a significant effect of genotype in male but not female rats in ES50 [males *p* < 0.0001, *F*(3, 27) = 14.76; females *p* = 0.1610, *F*(3, 33) = 1.829]. Again, whereas all male *Cntnap2* KO showed a lower ES50 than WT males (*Cntnap2* KO^het^ *p* = 0.0001, *Cntnap2* KO^hom^ *p* < 0.0001, *Cntnap2* KO^CF^ *p* = 0.0333), male *Cntnap2* KO^CF^ rats showed a significantly higher ES50 than male *Cntnap2* KO^hom^ rats (*p* = 0.0211; Figures 3C,F). For saturation, there was no effect of genotype for males or females [males *p* = 0.0979, *F*(3, 27) = 2.318; females *p* = 0.0696, *F*(3, 33) = 2.587; Figures 3C,G]. Lastly, there was a significant effect of genotype on the slope of the startle I/O function in males [*p* = 0.0056, *F*(3, 27) = 5.240] and females [*p* = 0.0291, *F*(3, 33) = 3.399]. Both *Cntnap2* KO^hom^ males and females showed lower slopes than their WT counterparts (males *p* = 0.0026, females *p* = 0.0207; Figure 3D).

Taken together, we found that male *Cntnap2* KO^CF^ compared to male *Cntnap2* KO^hom^ exhibited a less exaggerated startle response as evidenced by a decreased maximum response (*Top*) as well as a less decreased threshold and ES50 of the startle response I/O curves. Cross-fostering does not appear to have affected the startle response of *Cntnap2* KO females.

### Cross-fostering improves PPI deficit in *Cntnap2* KO rats

**Figure 4:**
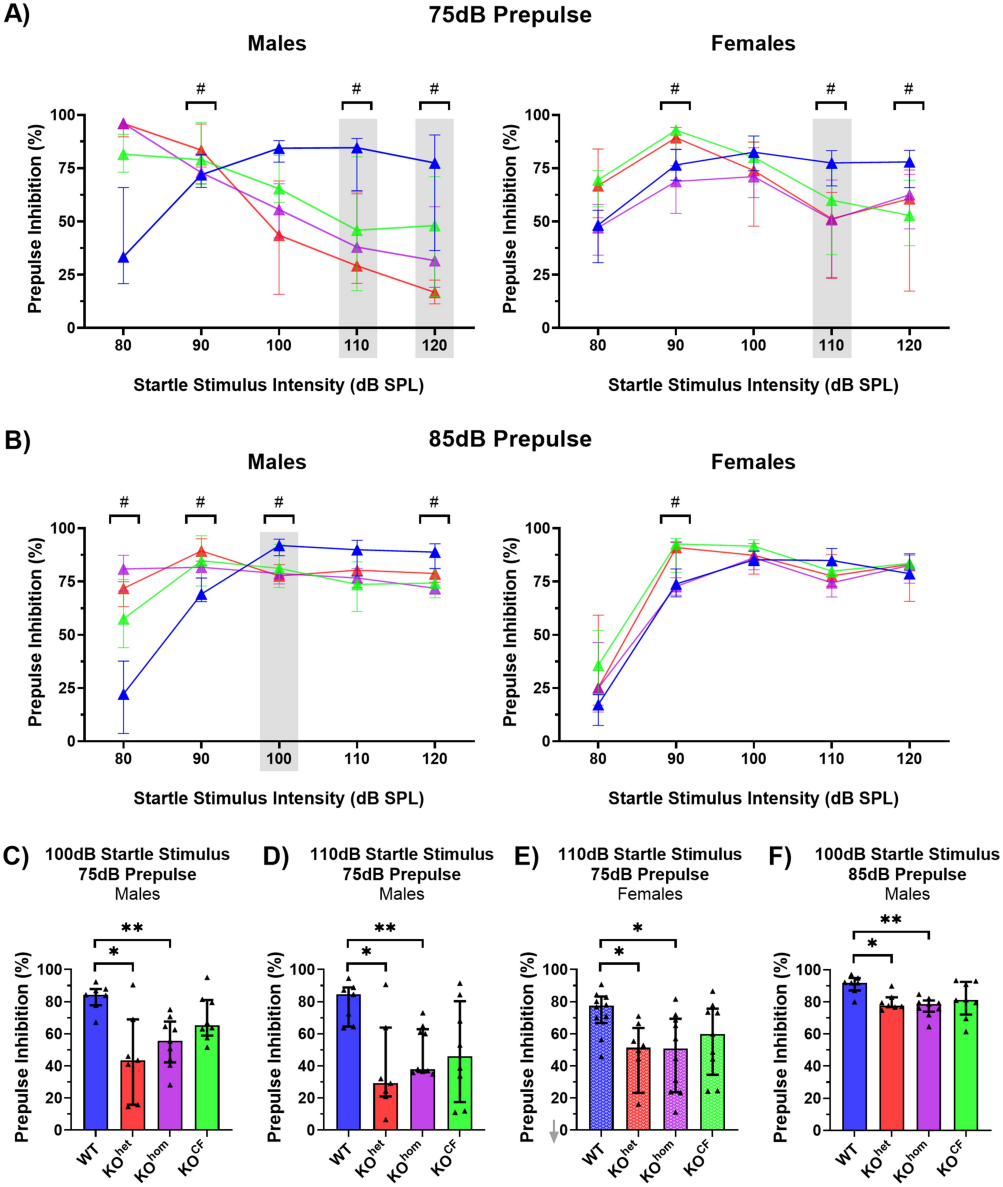
Cross-fostered *Cntnap2* knockout males and females show improved PPI. **(A)** PPI (% inhibition) across startle stimulus intensity levels with the 75 dB prepulse. # denotes startle stimulus intensities where there were group differences in PPI. Stimulus levels highlighted by gray rectangles indicate significant improvements by cross-fostering and are presented in the bottom panel. **(B)** PPI analysis across startle stimulus intensity with the 85 dB prepulse. **(C)** PPI of males with 100 dB startle stimulus with a 75 dB prepulse. PPI in male *Cntnap2* KO^CF^ is not different from WT. **(D)** PPI in males at the 110 dB startle stimulus with a 75 dB prepulse. Again, PPI of male *Cntnap2* KO^CF^ is not different from WT. **(E)** PPI of females at the 110 dB startle stimulus with a 75 dB prepulse. PPI of *Cntnap2* KO^CF^ females is not different from WT females. **(F)** PPI of males at the 100 dB startle stimulus with an 85 dB prepulse. PPI of *Cntnap2* KO^CF^ males is not different from WT males. *p < 0.05, **p < 0.01, ***p < 0.0001.

PPI was assessed across the entire range of startle stimulus levels (70 dB, 80 dB, 90 dB, 100 dB, 110 dB, and 120 dB) with a 75 dB and 85 dB prepulse (Figures 4A,B). With a 75 dB prepulse, there was an interaction effect between sex and startle stimulus level and between sex, genotype, and startle stimulus level [sex × startle stimulus *p* < 0.0001, *F*(5, 300) = 14.29; sex × genotype × startle stimulus *p* = 0.0013, *F*(15, 300) = 2.554]. With an 85 dB prepulse, these same interaction effects were found as well as a significant effect of sex and an interaction effect between sex and genotype [sex *p* < 0.0001, *F*(1, 60) = 286.9; sex × genotype *p* < 0.0001, *F*(3, 60) = 162.0; sex × startle stimulus *p* < 0.0001, *F*(5, 300) = 95.79; sex × genotype × startle stimulus *p* < 0.0001, *F*(15, 300) = 32.64]. Therefore, for both prepulse levels, males and females were separated for further analysis.

With a 75 dB prepulse, there was a significant effect of startle stimulus level and an interaction effect of genotype and startle stimulus level for males [startle stimulus level *p* < 0.0001, *F*(5, 135) = 33.89; genotype × startle stimulus *p* < 0.0001, *F*(15, 135) = 7.992] and females [startle stimulus level *p* < 0.0001, *F*(5, 165) = 43.22; genotype × startle stimulus *p* < 0.0001, *F*(15, 135) = 3.370; Figure 4A]. *Post-hoc* tests revealed PPI differences in males at the 80 dB, 100 dB, and 110 dB startle stimulus. At the 80 dB stimulus, all *Cntnap2* KO males showed greater PPI than WT males (*Cntnap2* KO^het^ *p* < 0.0001, *Cntnap2* KO^hom^ *p* = 0.0002, *Cntnap2* KO^CF^ *p* = 0.0011). Interestingly, whereas *Cntnap2* KO^het^ and KO^hom^ males exhibited lower PPI than WT males at the 100 dB stimulus (*Cntnap2* KO^het^ *p* = 0.0337, *Cntnap2* KO^hom^ *p* = 0.0009) and 110 dB stimulus (*Cntnap2* KO^het^ *p* = 0.0425, *Cntnap2* KO^hom^ *p* = 0.0077), PPI in KO^CF^ males did not differ from WT males (100 dB *p* = 0.2216, 110 dB *p* = 0.1439; Figures 4C,D).

For females with a 75 dB prepulse, similar effects were found at the 110 dB startle stimulus: whereas *Cntnap2* KO^het^ and KO^hom^ females showed lower PPI than WT females (*Cntnap2* KO^het^ *p* = 0.0112, *Cntnap2* KO^hom^ *p* = 0.0399), PPI in KO^CF^ females did not differ from WT females (*p* = 0.3114; Figure 4E). *Cntnap2* KO^CF^ females also showed greater PPI than WT at the 80 dB startle stimulus (*p* = 0.0039), but lower PPI at the 120 dB stimulus (*p* = 0.0465).

With an 85 dB prepulse, there was a main effect of genotype and startle stimulus intensity, as well as an interaction effect for males [genotype *p* < 0.0001, *F*(3, 27) = 55.97; startle stimulus level *p* < 0.0001, *F*(5, 135) = 27.32; genotype × startle stimulus *p* < 0.0001, *F*(15, 135) = 12.90] and females [genotype *p* = 0.0033, *F*(3, 33) = 5.563; startle stimulus level *p* < 0.0001, *F*(5, 165) = 92/57; genotype × startle stimulus *p* = 0.0013, *F*(15, 165) = 2.644; Figure 4B]. Similarly to what was observed with the 75 dB prepulse, at the 100 dB startle stimulus *Cntnap2* KO^het^ and KO^hom^ males showed lower PPI than WT males (*Cntnap2* KO^het^ *p* = 0.0116, *Cntnap2* KO^hom^ *p* = 0.0021), but KO^CF^ males did not differ from WT males (*p* = 0.2259; Figure 4F). With the 120 dB startle stimulus, *Cntnap2* KO^hom^ but not KO^CF^ males had a lower PPI than WT males (*Cntnap2* KO^hom^ *p* = 0.0252, *Cntnap2* KO^CF^ *p* = 0.2206). With the 85 dB prepulse, females only differed at the 90 dB stimulus, where *Cntnap2* KO^CF^ females exhibited even greater PPI than WT females (*p* = 0.0177).

In summary, cross-fostering showed positive effects on PPI at both prepulse intensities for males and females. Cross-fostered males and females exhibited ameliorated PPI impairment at the higher startle stimulus intensities. In addition, *Cntnap2* KO^CF^ females showed even better PPI than WT animals at lower startle stimulus intensities.

We further examined how cross-fostering affects PPI by assessing how the baseline I/O curve changes with adding a prepulse. As described by Doornaert et al. and El-Cheikh Mohamad et al., the entire baseline I/O curve can be scaled by two different components: startle and sound scaling (2023; 2024). Startle scaling, as shown by a downward shift in the I/O curve, results from a reduction in response amplitude, whereas sound scaling, as shown by a rightward shift in the I/O curve, results from a reduction in sound sensitivity. Like our previous study, here we show that *Cntnap2* KO rats have intact startle scaling but altered sound scaling compared to WT rats (Doornaert et al., 2024). However, *Cntnap2* KO^CF^ animals did not differ significantly from KO^hom^ animals in our measures of startle and sound scaling (see Supplementary Figures 7, 8). Therefore, we cannot use our differentiation of startle and sound scale components to explain the improved PPI observed in *Cntnap2* KO^CF^.

### Cross-fostering does not alleviate the gap-PPI impairment in *Cntnap2* KO rats; rather, it may exacerbate the impairment

**Figure 5:**
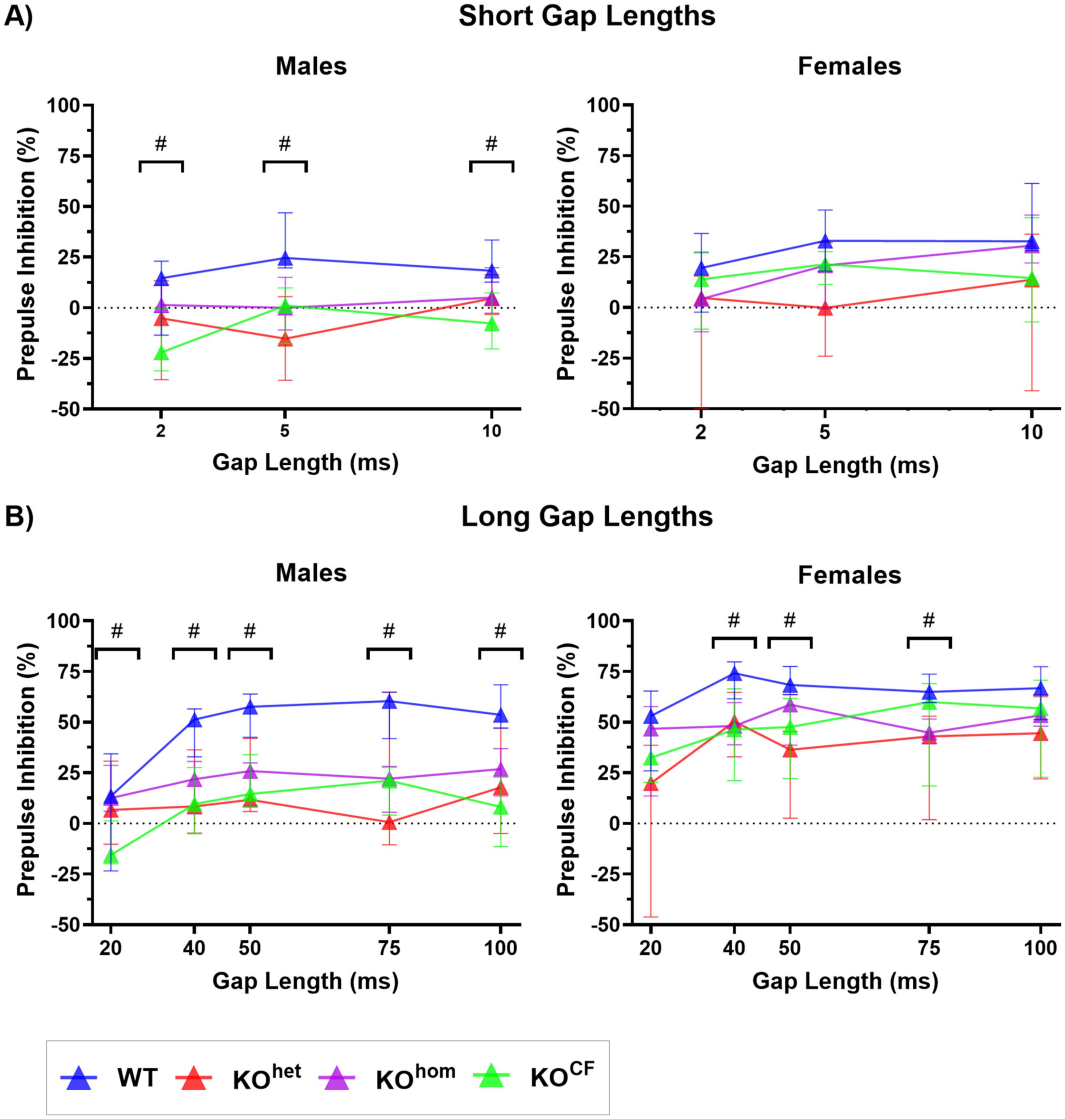
Gap-PPI impairment found in *Cntnap2* knockout rats is not improved by cross-fostering. **(A)** PPI (% inhibition) with short gap lengths (2 ms, 5 ms, and 10 ms). Male *Cntnap2* KO^hom^ showed a lower PPI with a 5 ms gap than WT rats, whereas KO^CF^ rats had a lower %PPI than WT rats at all short gap lengths. PPI did not differ between female *Cntnap2* WT and KO rats for short gap lengths. **(B)** PPI with long gap lengths (20 ms, 40 ms, 50 ms, 75 ms, and 100 ms). All *Cntnap2* KO males had a lower PPI than WT males for gap lengths between 40 ms and 100 ms. At 20 ms, *Cntnap2* KO^CF^ males had a lower PPI than KO^hom^ males. *Cntnap2* KO^hom^ and KO^CF^ females had lower PPI than WT females with a 40 ms gap length. Female *Cntnap2* KO^CF^ but not KO^hom^ showed a lower PPI than WT with a 50 ms gap length, and female *Cntnap2* KO^hom^ but not KO^CF^ had a lower PPI than WT with a 75 ms gap length. # denotes gap lengths where there were group differences in PPI.

Gap-PPI was assessed using small gaps in the background noise (2 ms, 5 ms, and 10 ms; Figure 5A) and long gap durations (20 ms, 40 ms, 60 ms, 75 ms, and 100 ms; Figure 5B). For small and long gaps, there was a significant effect of sex on PPI, and therefore, males and females were separated for further analysis [short *p* = 0.0100, *F*(1, 66) = 7.031; long *p* < 0.0001, *F*(1, 66) = 39.99].

For the short gaps, there was a significant effect of genotype and gap length on PPI in males [genotype *p* = 0.0002, *F*(3, 35) = 8.533; gap length *p* = 0.0134, *F*(2, 70) = 4.591]. Only male *Cntnap2* KO^CF^ rats had a lower PPI than WT males with a 2 ms and 10 ms gap (2 ms *p* = 0.0360, 10 ms *p* = 0.0062; Figure 5A), whereas with a 5 ms gap, all male KO rats regardless of group showed lower PPI than WT males (*Cntnap2* KO^het^ *p* = 0.0017, *Cntnap2* KO^hom^ *p* = 0.0140, *Cntnap2* KO^CF^ *p* = 0.0191). Although females also had a significant effect of genotype and gap length on PPI for short gaps, there were no significant differences found between groups following *post-hoc* testing [genotype *p* = 0.0481, *F*(3, 31) = 2.948; gap length *p* = 0.0197, *F*(2, 62) = 4.186].

For long gaps, there was a significant effect of genotype and gap length on PPI, as well as an interaction effect between genotype and gap length [genotype *p* = 0.0003, *F*(3, 35) = 8.333; gap length *p* < 0.0001, *F*(4, 140) = 12.23; genotype × gap length *p* = 0.0053, *F*(12, 140) = 2.502]. For the 20 ms gap, male *Cntnap2* KO^CF^ rats showed lower PPI than male WT animals (*p* = 0.0139; Figure 5B). At all longer gap lengths, all male *Cntnap2* KO rats regardless of group had lower PPI than WT males (40 ms: *Cntnap2* KO^het^ *p* = 0.0498, *Cntnap2* KO^hom^ *p* = 0.0198, *Cntnap2* KO^CF^ *p* = 0.0117; 50 ms: *Cntnap2* KO^het^ *p* = 0.0207, *Cntnap2* KO^hom^ *p* = 0.0076, *Cntnap2* KO^CF^ *p* = 0.0028; 75 ms: *Cntnap2* KO^het^ *p* = 0.0259, *Cntnap2* KO^hom^ *p* = 0.0015, *Cntnap2* KO^CF^ *p* = 0.0014; 100 ms: *Cntnap2* KO^het^ *p* = 0.0418, *Cntnap2* KO^hom^ *p* = 0.0206, *Cntnap2* KO^CF^ *p* = 0.0011). For females, there was again a significant effect of genotype and gap length on PPI [genotype *p* = 0.0059, *F*(3, 31) = 5.028; gap length *p* = 0.0003, *F*(4, 124) = 5.66]. With a 40 ms gap, female *Cntnap2* KO^hom^ and KO^CF^ rats had lower PPI than WT rats (*Cntnap2* KO^hom^ *p* = 0.0102, *Cntnap2* KO^CF^ *p* = 0.0315). With a 50 ms gap length, *Cntnap2* KO^CF^ but not KO^hom^ showed lower PI than female WT rats (*Cntnap2* KO^hom^ *p* = 0.1098, *Cntnap2* KO^CF^ *p* = 0.0127). In contrast, *Cntnap2* KO^hom^, but not KO^CF^, had a lower PPI than WT females with a 75 ms gap (*Cntnap2* KO^hom^ *p* = 0.0008, *Cntnap2* KO^CF^ *p* = 0.2151). Overall, it appears that cross-fostering did not reverse the gap-PPI impairment observed in *Cntnap2* KO rats but rather exacerbated the deficit at certain gap lengths.

## Discussion

This study investigated the impact of early postnatal environmental factors on long-term auditory processing and sensorimotor gating in the *Cntnap2* KO rat model. *Cntnap2* KO^CF^ rats showed partial restoration of auditory maturation, evidenced by improved hearing sensitivity, a less exaggerated startle response, and enhanced PPI compared to *Cntnap2* KO^hom^ rats. These results indicate that early environmental interventions can positively modulate some deficits associated with the loss-of-function of *Cntnap2*. Despite these improvements, *Cntnap2* KO^CF^ rats also displayed more pronounced changes in the ABR, including reduced neural responsiveness and slower conduction times, along with persistent gap-PPI impairments. These findings highlight that while the early postnatal environment can modify certain effects of the loss of *Cntnap2*, it may also exacerbate other deficits, illustrating the complex nature of gene–environment interactions in neurodevelopment.

### Cross-fostering has both beneficial and consequential effects on ABR

Hearing sensitivity, measured by the threshold to a click stimulus, typically increases from the postnatal period to adulthood as the auditory pathway matures (Smith and Kraus, 1987; Hang et al., 2016). Notably, this maturation of hearing sensitivity was not observed in *Cntnap2* KO^hom^ animals but was restored in KO^CF^ animals. While the ABR threshold reflects overall auditory pathway function, wave amplitudes and latencies provide more insights into neural activity and conduction. Adult *Cntnap2* KO^CF^ animals exhibited smaller wave I amplitudes than WT, suggesting reduced auditory nerve activity, and slower interpeak latencies, indicating impaired conduction between brainstem regions. Hence, cross-fostering reversed hearing sensitivity deficits, but neural responsiveness and conduction of the auditory brainstem remained impaired.

*Cntnap2* plays an important role in neural development, myelination, and synaptic function, which is important particularly in the development of pathways requiring precise timing (Truong et al., 2015; Gordon et al., 2016; Poot, 2017). Cross-fostering during the critical period exposes *Cntnap2* KO animals to an enriched sensory/auditory environment, likely promoting neural plasticity, and potentially improving ABR thresholds through mechanisms like synaptic upscaling or increased neurotransmitter release in central auditory structures. However, the lower wave I amplitude suggests that these brainstem and/or cortical compensatory mechanisms may strain peripheral auditory nerve function perhaps through increased efferent feedback (Boothalingam et al., 2023). The slower interpeak latencies in our *Cntnap2* KO animals reflect impaired synaptic transmission and/or myelination, a common observation in *Cntnap2* KO models (Varea et al., 2015; Scott et al., 2019, 2022). These neural conduction deficits persisted despite cross-fostering, suggesting that environmental factors cannot fully compensate for these *Cntnap2*-related impairments.

The finding that cross-fostered animals exhibited greater ABR disruptions, particularly reduced wave I amplitude and prolonged wave I–IV interpeak latency, suggests that cross-fostering exerts a stronger influence on central auditory plasticity than on peripheral conduction integrity. The reduced wave I amplitude indicates diminished auditory nerve responsiveness, possibly due to increased efferent modulation from central auditory structures, such as the medial olivocochlear system, which regulates peripheral auditory excitability (Boothalingam et al., 2023). This could reflect a compensatory mechanism aimed at reducing auditory hyperexcitability at higher processing levels. However, the persistent delays in interpeak latencies suggest that environmental enrichment is insufficient to overcome myelination-dependent deficits in synaptic transmission and conduction velocity within the auditory brainstem. While cross-fostering may enhance auditory plasticity by altering central processing, it does not fully restore the timing-dependent aspects of auditory function that rely on intact myelination.

### Cross-fostering reduces heightened startle in *Cntnap2* KO males and improves PPI deficit in *Cntnap2* KO males and females

Cross-fostering alleviated increased startle responses and PPI deficits in *Cntnap2* KO rats, with improvements in startle responses found in males and in PPI observed in both males and females. These changes likely result from enhanced neural plasticity during the critical period of auditory development. The startle response is regulated by a well-characterized brainstem pathway involving cochlear root neurons, the caudal pontine reticular nucleus (PnC), and spinal cord motor neurons (for review see Koch, 1999; Zheng and Schmid, 2023). PPI is thought to be mediated by a feed-forward inhibitory circuit starting with cochlear neurons projecting to the inferior colliculus and the pedunculopontine tegmental nucleus (PPTg; Koch, 1999). *Cntnap2* is prominently expressed in the neural circuits governing startle and PPI, and its dysfunction likely disrupts the balance of excitatory and inhibitory signaling in these pathways (Gordon et al., 2016; Scott et al., 2018). Previous studies have shown elevated levels of excitatory and inhibitory neurotransmitters in the PnC of *Cntnap2* KO rats and altered firing rates in response to startling sounds that likely account for the increased startle (Möhrle et al., 2021; Zheng et al., 2023). Besides these changes in the primary startle pathway, minimal differences were observed in PPTg firing rates and PnC inhibition from the PPTg in *Cntnap2* KO rats, indicating that these structures do not account for the reduced PPI (Zheng et al., 2023). Cross-fostering may mitigate increased startle and impaired PPI by decreasing PnC hyperexcitability due to enriched sensory input during the critical period, possibly through increased GABAergic signaling or reduced glutamatergic activity. Similar effects have been observed with pharmacological treatments like R-Baclofen, which dampens hyperexcitability in the startle pathway (Möhrle et al., 2021). Cross-fostering may also induce structural changes in the PnC, such as increased dendritic branching, enhanced spine density, and improved myelination, all of which are influenced by *Cntnap2* expression (Anderson et al., 2012; Scott et al., 2019).

The observed sex differences in startle response and PPI in *Cntnap2* KO rats may be attributed to distinct underlying neural mechanisms. Prior research suggests that heightened startle in male and female *Cntnap2* KO rats arises from different factors: males exhibit both increased startle magnitude and a leftward shift in the startle I/O function, indicating heightened sound sensitivity, whereas females show only increased startle magnitude (Doornaert et al., 2024). *In vivo* electrophysiological recordings further support this distinction, as female *Cntnap2* KO rats display increased firing rates of PnC neurons in response to startle stimuli, while males exhibit enhanced recruitment of startle-mediating neurons, potentially accounting for their increased sound sensitivity (Zheng et al., 2023, 2024). Given these sex-specific differences, cross-fostering may impact males and females differently by modulating PnC hyperexcitability through distinct pathways. For instance, in males, cross-fostering could reduce the recruitment of startle-mediating neurons, thereby normalizing the leftward shift in the I/O function, while in females, it may directly attenuate PnC hyperactivity, reducing startle magnitude.

These findings align with studies of the effects of environmental enrichment on startle and PPI, which have shown beneficial effects in the valproic acid rat model and mGlur5 KO mice (Schneider et al., 2006; Burrows et al., 2015). Cross-fostering may act as a form of environmental enrichment by providing increased maternal care, exposure to social ultrasonic vocalizations, and varied social interactions with littermates. These changes could modulate sensory processing in *Cntnap2* KO rats and improve the function of startle and PPI networks. Future studies will address the effect of housing *Cntnap2* KO rats in sensory-enriched environments during early development on sensory processing.

### Gap-PPI deficit revealed in *Cntnap2* KO rat; not improved by cross-fostering

This study is the first to evaluate gap-PPI in the *Cntnap2* KO rat, which assesses an animal’s ability to perceive and respond to brief gaps in continuous noise, reflecting auditory temporal resolution (Fournier and Hébert, 2016). This capability is crucial for speech and sound processing, especially in distinguishing rapid changes in sound patterns (Poldrack et al., 2001). Unlike traditional PPI, gap-PPI involves greater engagement of the primary auditory cortex (Bowen et al., 2003; Weible et al., 2014; Moreno-Paublete et al., 2017). While gap-PPI has often been used to assess tinnitus (Galazyuk and Hébert, 2015), recent findings suggest it may also serve as a biomarker for ASD (Foss-Feig et al., 2018). In fact, *Cntnap2* KO mice have shown deficits in gap-PPI, specifically exhibiting a significantly higher detection threshold for short gaps compared to control mice (Truong et al., 2015). We observed a similar pattern of impairment in *Cntnap2* KO rats, with short gaps affected primarily in males and longer gaps impaired in both males and females. These deficits are likely due to immature and hyperexcitable auditory cortex neurons (Scott et al., 2022), which may elevate “neuronal noise,” making it harder to distinguish relevant stimuli from background noise.

Cross-fostering did not improve gap-PPI and even exacerbated impairments at certain gap lengths. This suggests that gap-PPI, which relies heavily on cortical processing (Bowen et al., 2003; Weible et al., 2014; Moreno-Paublete et al., 2017), may not benefit from cross-fostering, unlike startle and prepulse inhibition, which are largely brainstem-dependent (Wright and Barnes, 1972; Hammond, 1974; Davis and Gendelman, 1977; Hunter and Willott, 1993). The finding that cross-fostering exacerbated gap-PPI deficits highlights the complexity of gene–environment interactions in auditory processing. Given that early environmental interventions can influence neurodevelopment by altering sensory input and plasticity, it is possible that increased environmental stimulation further disrupted auditory cortical processing rather than normalizing it. This could be due to excessive neural excitability in the auditory cortex of *Cntnap2* KO rats (Scott et al., 2022; Mann et al., 2023) where additional sensory input amplifies rather than mitigates cortical dysregulation. Alternatively, cross-fostering might have affected the development of inhibitory circuits in a way that selectively worsened temporal resolution deficits. Future studies examining cortical excitability, inhibitory signaling, and synaptic plasticity in the auditory cortex following cross-fostering could help elucidate the mechanisms underlying this unexpected outcome.

### Limitations and future directions

This study is limited in its ability to identify the specific environmental factors being manipulated by the cross-fostering paradigm. As noted earlier, variations in maternal care may exist between *Cntnap2* KO and HET dams. Maternal behaviors, such as grooming and milk quality, are known to significantly impact pup development (Champagne et al., 2003; Qiu et al., 2020; DeRosa et al., 2022). Another factor to consider is the genetic composition of the litter. While homozygous breeding results in exclusively homozygous offspring, heterozygous breeding produces a mix of homozygous, heterozygous, and wildtype pups. Consequently, cross-fostered animals are exposed to different social environments, which could influence social communication and interactions, ultimately shaping their developmental outcomes. Moreover, the process of cross-fostering could induce stress on the pups, again potentially affecting development (Vorhees et al., 2024). Notwithstanding the challenges of identifying the crucial factors, our findings demonstrate that environmental influences induced by cross-fostering can significantly alter developmental outcomes regarding auditory brainstem processing.

Another limitation of this study is the one-way cross-fostering design as we did not cross-foster *Cntnap2* KO pups bred from a *Cntnap2* HET dam to a KO dam. Cross-fostering was limited to litters born within 48 h to align with the critical period for early auditory development. Given the need for many more litters to be born within this period and the low reproductive success rate of homozygous KO dams, implementing reciprocal cross-fostering was impractical. As a result, while the benefits observed in the heterozygous environment are noted, the potential positive, negative, or neutral impacts of the homozygous environment on sensory processing remain undetermined.

Future research should investigate prenatal factors that may differ between heterozygous and homozygous pairings. Variations in dam health, including hormonal, metabolic, and stress responses, could influence fetal development and brain function (Fatima et al., 2017; Baud and Berkane, 2019). Homozygous KO dams may experience distinct prenatal environments, such as altered uterine blood flow, oxygen levels, and placental function, which could impact critical physiological processes during pregnancy (Hufnagel et al., 2022). Additionally, maternal mitochondrial DNA (mtDNA), which is inherited maternally, could play a role in neurodevelopmental outcomes, especially since mitochondrial dysfunction has been repeatedly linked to autism (Wang et al., 2022; Al-Kafaji et al., 2023). Lastly, investigating the role of parental vocalizations during the critical period could further enhance understanding of how early auditory experiences, such as exposure to biological parental sounds during cross-fostering, shape sensory processing and developmental outcomes in ASD models.

## Data Availability

The original contributions presented in the study are included in the article/Supplementary material, further inquiries can be directed to the corresponding author.
