# Supplementary material for: Postnatal environment affects auditory development and sensorimotor gating in a rat model for autism spectrum disorder
Source: Front Neurosci. 2025 Mar 11;19:1565919. doi: 10.3389/fnins.2025.1565919 (PMC11933038; doi:10.3389/fnins.2025.1565919)
Supplement: Supplementary file 1 [file Data_Sheet_1.docx]

**
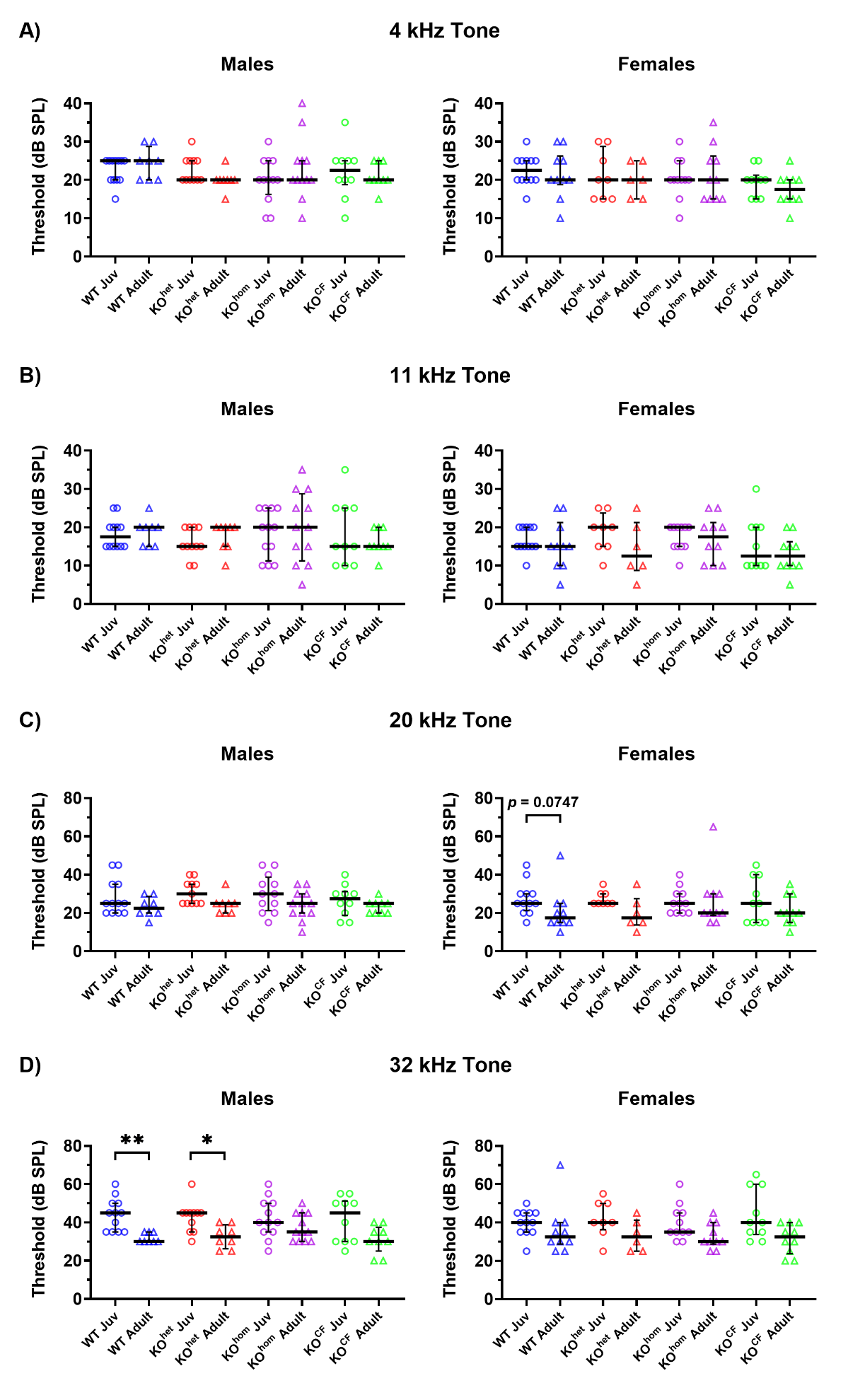
**

**Supplementary Figure 1.** Change in ABR threshold between juvenile age and adulthood for tone stimuli. **(A)** 4 kHz tone. There is no effect of genotype, age, or interaction effect of genotype and age on the threshold. **(B)** 11 kHz tone. There is no effect of genotype, age, or interaction effect of genotype and age on the threshold for males and females. **(C)** 20 kHz tone. There was a main effect of age on the threshold for males (age *p* = 0.0041, *F*(1, 33) = 9.501) and females (age *p* = 0.0202, *F*(1, 30) = 6.015). The threshold of *Cntnap2* WT females was trending towards being lower in adulthood compared to when they were juveniles (*p* = 0.0747). **(D)** 32 kHz tone. There was a main effect of age on the threshold for males (age *p* = 0.0002, *F*(1, 33) = 16.58) and females (age *p* < 0.0001, *F*(1, 30) = 13.72). *Cntnap2* WT and KO^het^ males showed lower thresholds in adulthood than when they were juveniles (*Cntnap2* WT *p* = 0.0035, *Cntnap2* KO^het^ *p* = 0.0215). *p < 0.05, **p < 0.01, ***p < 0.0001.


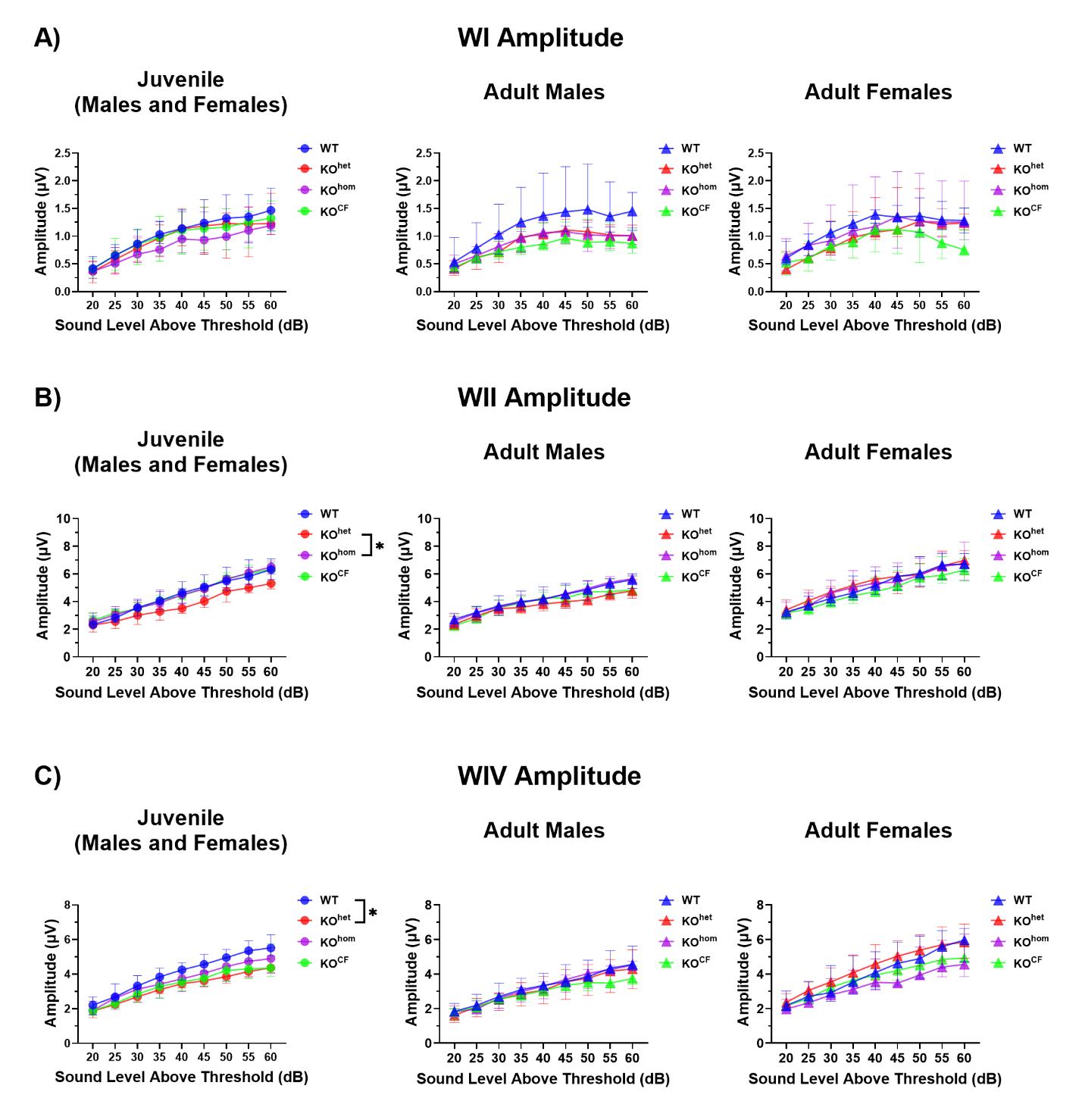


**Supplementary Figure 2.** ABR amplitude across sound levels. Subsequent analysis used peak amplitudes from 60 dB above the threshold. For juvenile measures, since there was no effect of sex or interaction effects involving sex, data was collapsed across sex. **(A)** WI peak amplitude in juvenile age and adulthood. There was no effect of genotype or interaction effect between genotype and sound level on peak amplitude across the sound levels at the juvenile age or adulthood. **(B)** WII peak amplitude in juvenile age and adulthood. There was a main effect of genotype and an interaction effect between genotype and sound level on juvenile peak amplitude (genotype *p* = 0.0293, *F*(3, 82) = 3.151, genotype × sound level *p* < 0.0001, *F*(24, 656) = 2.920). *Cntnap2* KO^het^ rats had a lower response amplitude than KO^hom^ rats (*p* = 0.0341). This did not persist into adulthood, as there was no effect of genotype or interaction effect between genotype and sound level on peak amplitude. **(C)** WIV peak amplitude in juvenile age and adulthood. There was a main effect of genotype and an interaction effect between genotype and sound level on juvenile peak amplitude (genotype *p* = 0.0181, *F*(3, 82) = 3.544, genotype × sound level *p* < 0.0001, *F*(24, 656) = 2.939). *Cntnap2* KO^het^ rats had a lower response amplitude than WT rats (*p* = 0.0393). This did not persist into adulthood, although there was a significant interaction effect between genotype and sound level for males (*p* = 0.0002, *F*(24, 264) = 2.547) and females (*p* = 0.0430, *F*(24, 256) = 1.590) on peak amplitude. Post-hoc tests did not reveal any group differences. *p < 0.05, **p < 0.01, ***p < 0.0001.


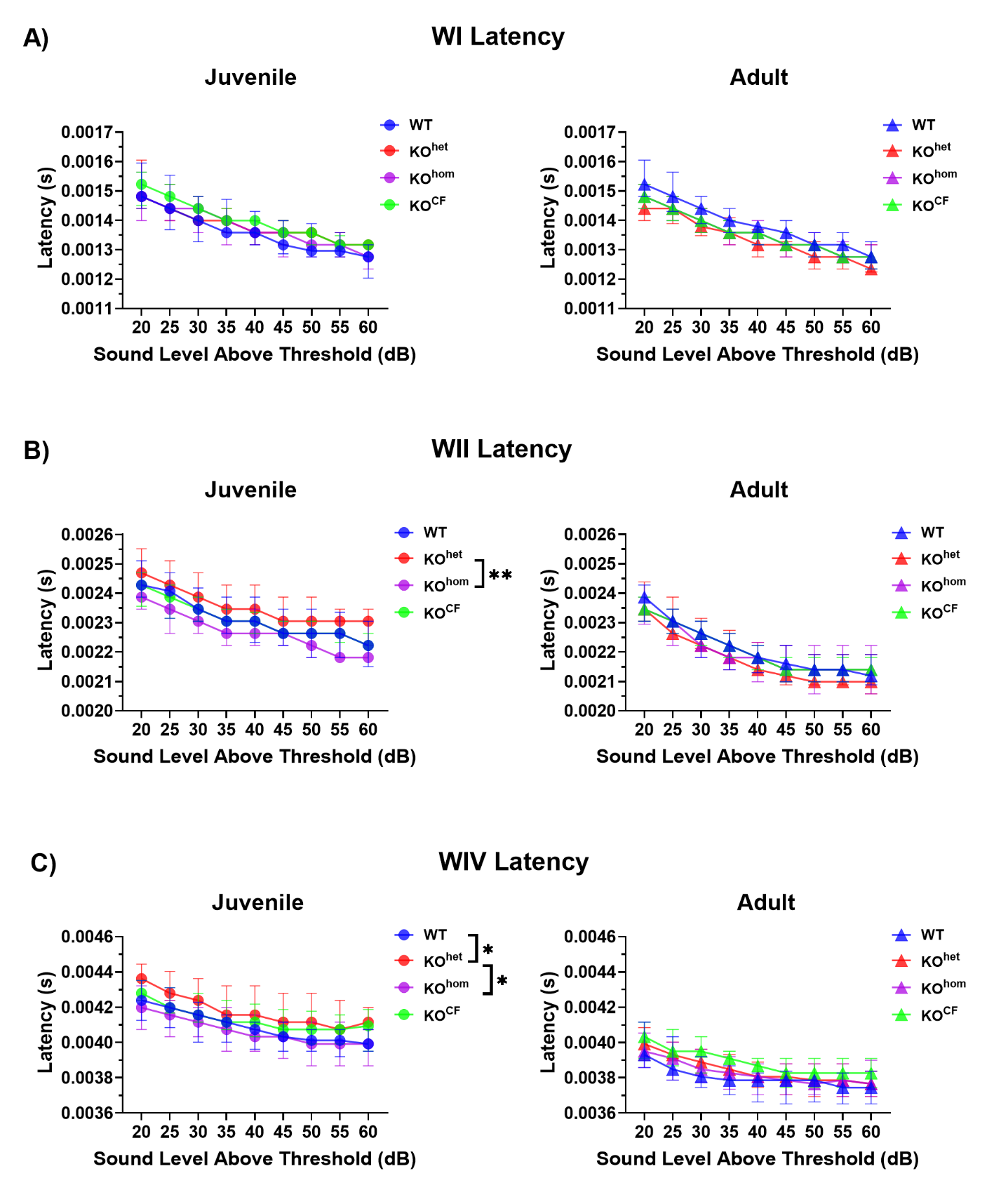


**Supplementary Figure 3.** ABR latencies across sound levels. Subsequent analysis used peak latencies from 60 dB above the threshold. For all measures, since there was no effect of sex or interaction effects involving sex, data was collapsed across sex. **(A)** WI peak latency in juvenile age and adulthood. There was no effect of genotype or interaction effect between genotype and sound level on peak latency across the sound levels at the juvenile age. In adulthood, there was an interaction effect between genotype and sound level on peak latency (*p* = 0.0228, *F*(24, 552) = 1.682), but post hoc tests revealed no differences between the groups. **(B)** WII peak latency in juvenile age and adulthood. There was a main effect of genotype and an interaction effect between genotype and sound level on juvenile peak latency (genotype *p* = 0.0132, *F*(3, 82) = 3.800, genotype × sound level *p* = 0.0022, *F*(24, 656) = 2.064). *Cntnap2* KO^het^ rats had a slower latency than KO^hom^ rats (*p* = 0.0056). This did not persist into adulthood, although there was a significant interaction effect between genotype and sound level for (*p* = 0.0005, *F*(24, 552) = 2.300) on peak latency. Post-hoc tests did not reveal any group differences. **(C)** WIV peak latency in juvenile age and adulthood. There was a main effect of genotype on juvenile peak latency (genotype *p* = 0.0105, *F*(3, 82) = 3.989). *Cntnap2* KO^het^ rats had a slower latency than WT and KO^hom^ rats (*Cntnap2* WT *p* = 0.0228, *Cntnap2* KO^hom^ *p* = 0.0172). This did not persist into adulthood, as there was no effect of genotype or interaction effect between genotype and sound level on peak latency. *p < 0.05, **p < 0.01, ***p < 0.0001.


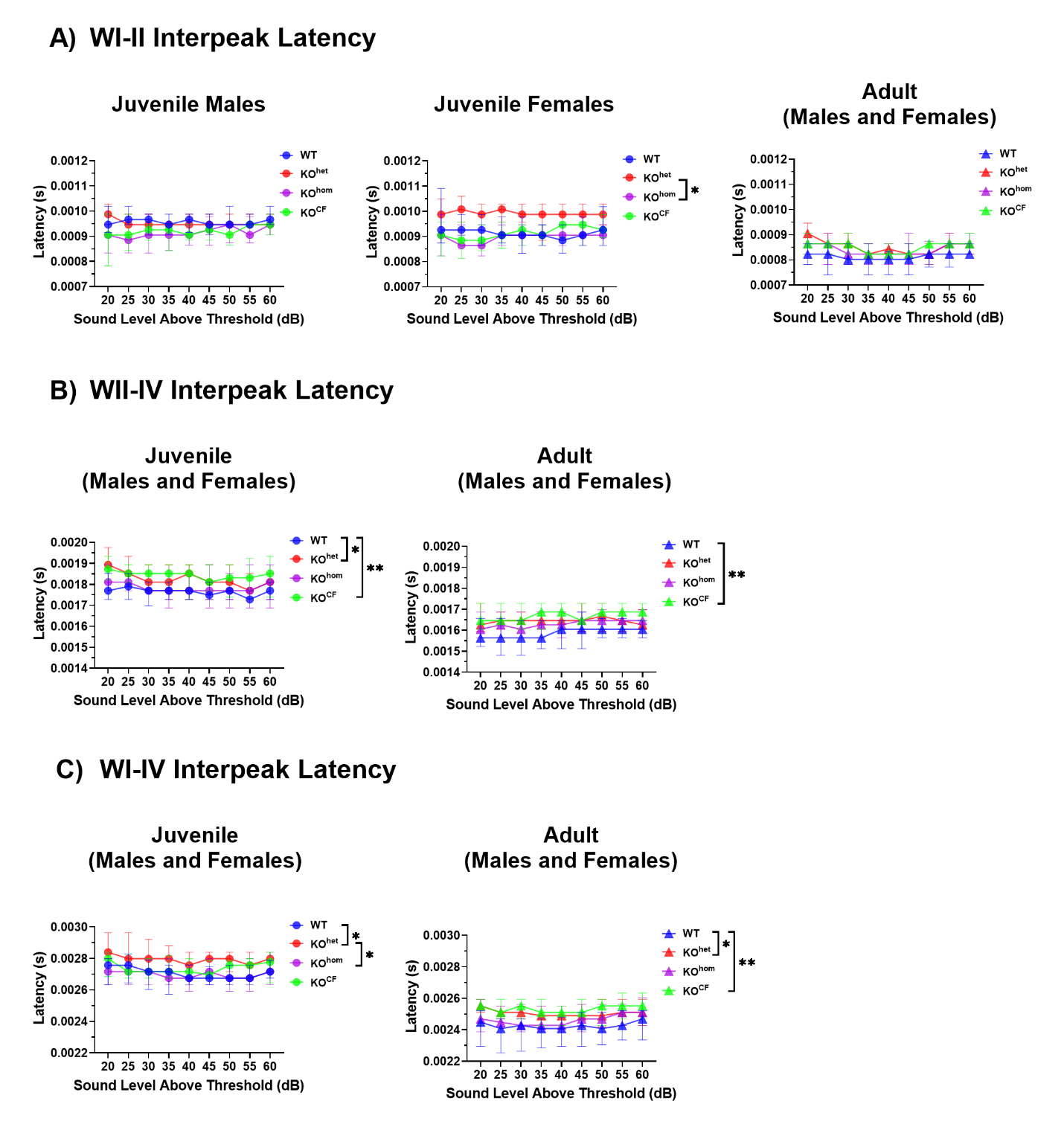


**Supplementary Figure 4.** ABR interpeak latencies across sound levels. Subsequent analysis used interpeak latencies from 60 dB above the threshold. For measures where there was no effect of sex or interaction effects involving sex, data was collapsed across sex. **(A)** WI-II interpeak latency in juvenile age and adulthood. There was an interaction effect between genotype and sound level on interpeak latency in juvenile females (*p* = 0.0339, *F*(24, 296) = 1.631). *Cntnap2* KO^het^ females had a slower interpeak latency than KO^hom^ females (*p* = 0.0473). In adulthood, there was a main effect of genotype and interaction effect between genotype and sound level on interpeak latency for all animals (genotype *p* = 0.0307, *F*(3, 69) = 3.142, genotype × sound level *p* = 0.0115, *F*(24, 552) = 1.802). However, post-hoc tests did not reveal any group differences. **(B)** WII-IV interpeak latency in juvenile age and adulthood. There was a main effect of genotype on interpeak latency at the juvenile age (*p* = 0.0065, *F*(3, 82) = 4.133). As juveniles, *Cntnap2* KO^het^ and KO^CF^ animals had slower interpeak latencies than WT animals (*Cntnap2* KO^het^ *p* = 0.0397, *Cntnap2* KO^CF^ *p* = 0.0079). The main effect of genotype on interpeak latency persisted in adulthood (*p* = 0.0030, *F*(3, 69) = 5.116). In adulthood, *Cntnap2* KO^CF^ animals had a slower interpeak latency than WT animals (*p* = 0.0017). **(C)** WI-IV interpeak latency in juvenile age and adulthood. There was a main effect of genotype on interpeak latency at the juvenile age (*p* = 0.0161, *F*(3, 82) = 3.641). As juveniles, *Cntnap2* KO^het^ animals had a slower interpeak latency than WT and KO^hom^ animals (*Cntnap2* WT *p* = 0.0170, *Cntnap2* KO^hom^ *p* = 0.0320). The main effect of genotype on interpeak latency persisted in adulthood, along with an interaction effect between genotype and sound level (genotype *p* = 0.0050, *F*(3, 69) = 4.661, genotype × sound level *p* = 0.0302, *F*(24, 552) = 1.632). In adulthood, *Cntnap2* KO^het^ and KO^CF^ animals had slower interpeak latencies than WT animals (*Cntnap2* KO^het^ *p* = 0.0279, *Cntnap2* KO^CF^ *p* = 0.0020). *p < 0.05, **p < 0.01, ***p < 0.0001.


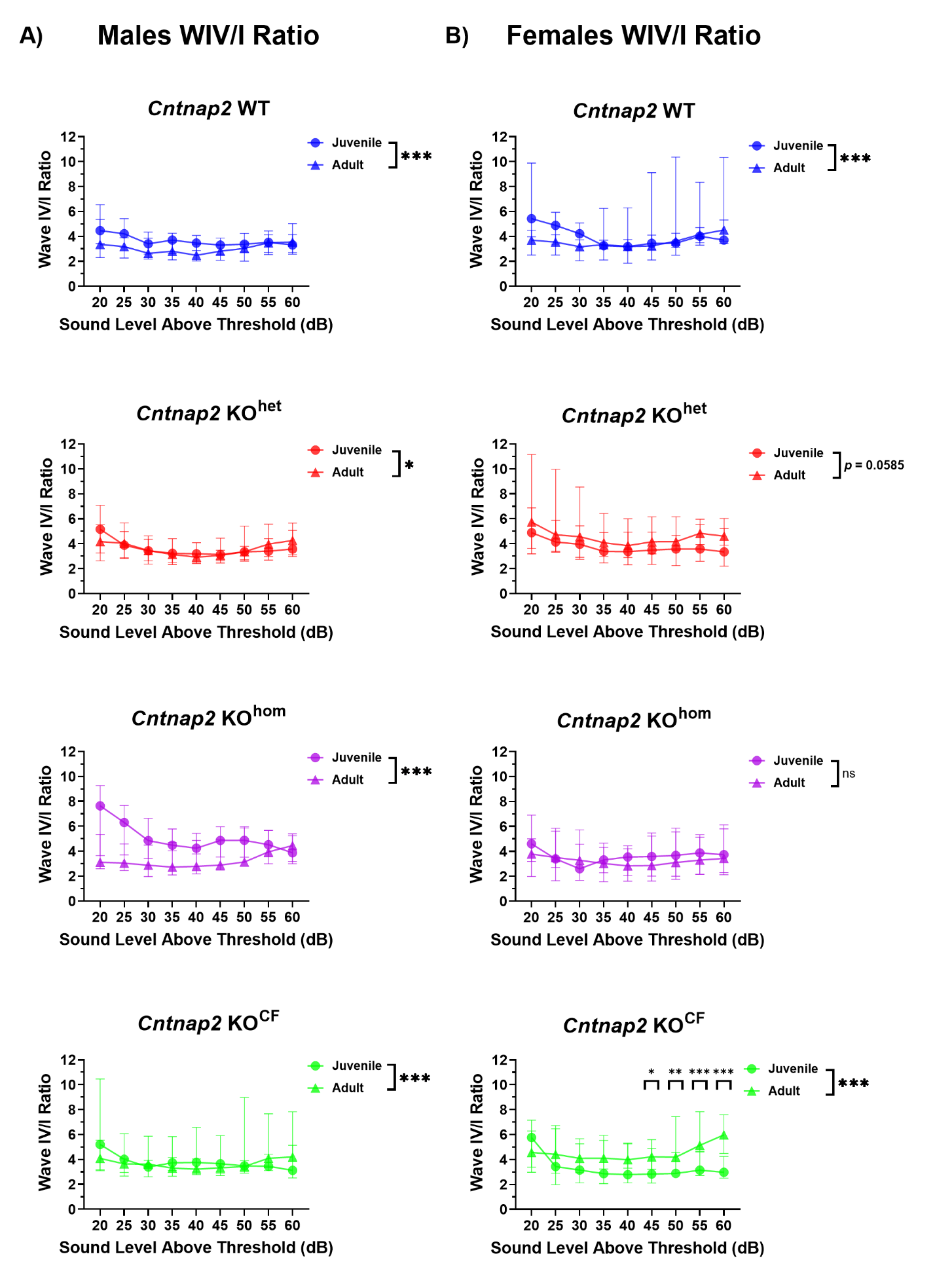


**Supplementary Figure 5.** ABR WIV/I ratio across sound levels. Subsequent analysis used WIV/I ratios from 60 dB above the threshold. **(A)** WIV/I ratio in juvenile and adult males. All groups showed a significant effect of age on the WIV/I ratio (*Cntnap2* WT *p* < 0.0001, *F*(1, 151) = 33.90, *Cntnap2* KO^het^ *p* = 0.0101, *F*(1, 143) = 6.802, *Cntnap2* KO^hom^ *p* < 0.0001, *F*(1, 187) = 28.80, *Cntnap2* KO^CF^ *p* < 0.0001, *F*(1, 144) = 232.0). However, post-hoc testing revealed no significant differences within groups between juvenile age and adulthood for all sound levels. **(B)** WIV/I ratio in juvenile and adult females. Only *Cntnap2* WT and KO^CF^ females had a significant effect of age on WIV/I ratio *Cntnap2* WT *p* < 0.0001, *F*(1, 169) = 243.7, *Cntnap2* KO^CF^ *p* < 0.0001, *F*(1, 153) = 66.02). There was no effect of age for *Cntnap2* KO^hom^ females, but the effect was trending towards significance for KO^het^ animals (*Cntnap2* KO^het^ *p* = 0.0585, *F*(1, 99) = 3.665, *Cntnap2* KO^hom^ *p* = 0.35517, *F*(1, 161) = 0.8598). Only *Cntnap2* KO^CF^ females showed significant differences between juvenile age and adulthood, with a greater adult ratio than the juvenile ratio at the higher sound intensities (45 dB *p* = 0.0122, 50 dB *p* = 0.0011, 55 dB *p* < 0.0001, 60 dB *p* < 0.0001). *p < 0.05, **p < 0.01, ***p < 0.0001, ns indicates non-significance of the comparison.


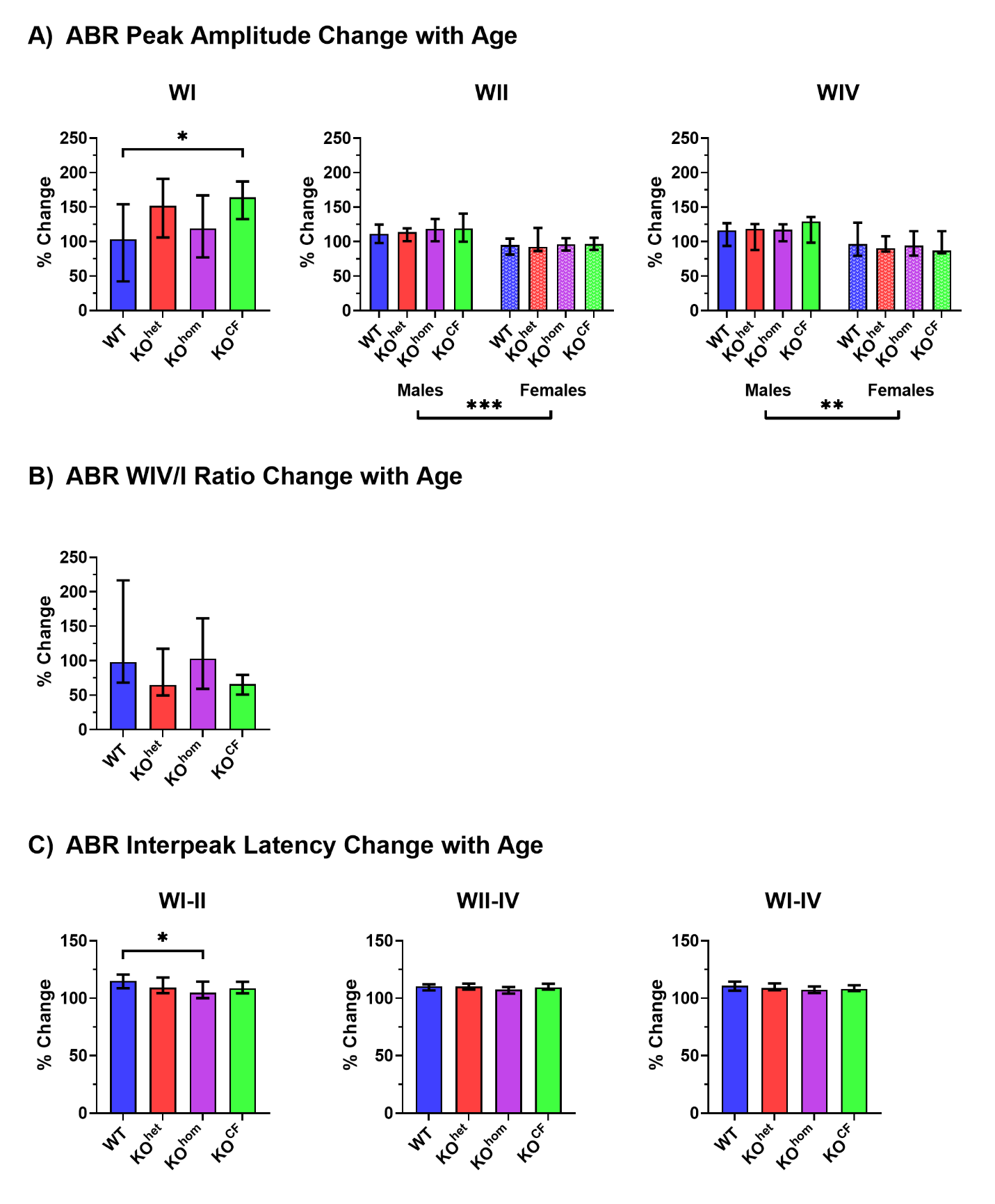


**Supplementary Figure 6.** ABR change in amplitude, WIV/I ratio, and interpeak latency with age at 60 dB above threshold. Ratios could only be calculated for animals that had juvenile and adult measures (*Cntnap2* WT: M = 8, F = 10; *Cntnap2* KO^het^: M = 8, F = 4; *Cntnap2* KO^hom^: M = 12, F = 10; *Cntnap2* KO^CF^: M = 9, F = 10). For measures where there was no effect of sex or interaction effects involving sex, data was collapsed across sex. **(A)** Percent change in amplitude from juvenile age to adulthood. A significant effect of genotype was only observed for WI (*p* = 0.0393, *F*(3, 63) = 2.951). *Cntnap2* KO^CF^ rats had a greater change in amplitude for WI than WT rats (*p* = 0.0434). **(B)** Percent change in WIV/I ratio from juvenile age to adulthood. There was no effect of genotype on the change in WIV/I ratio (*p* = 0.1742, *F*(3, 63) = 1.709). **(C)** Percent change in interpeak latency from juvenile age to adulthood. A significant effect of genotype was only observed for WI-II (*p* = 0.0439, *F*(3, 63) = 2.859). *Cntnap2* KO^hom^ rats showed a smaller change in WI-II interpeak latency than WT rats (*p* = 0.0388). *p < 0.05, **p < 0.01, ***p < 0.0001.


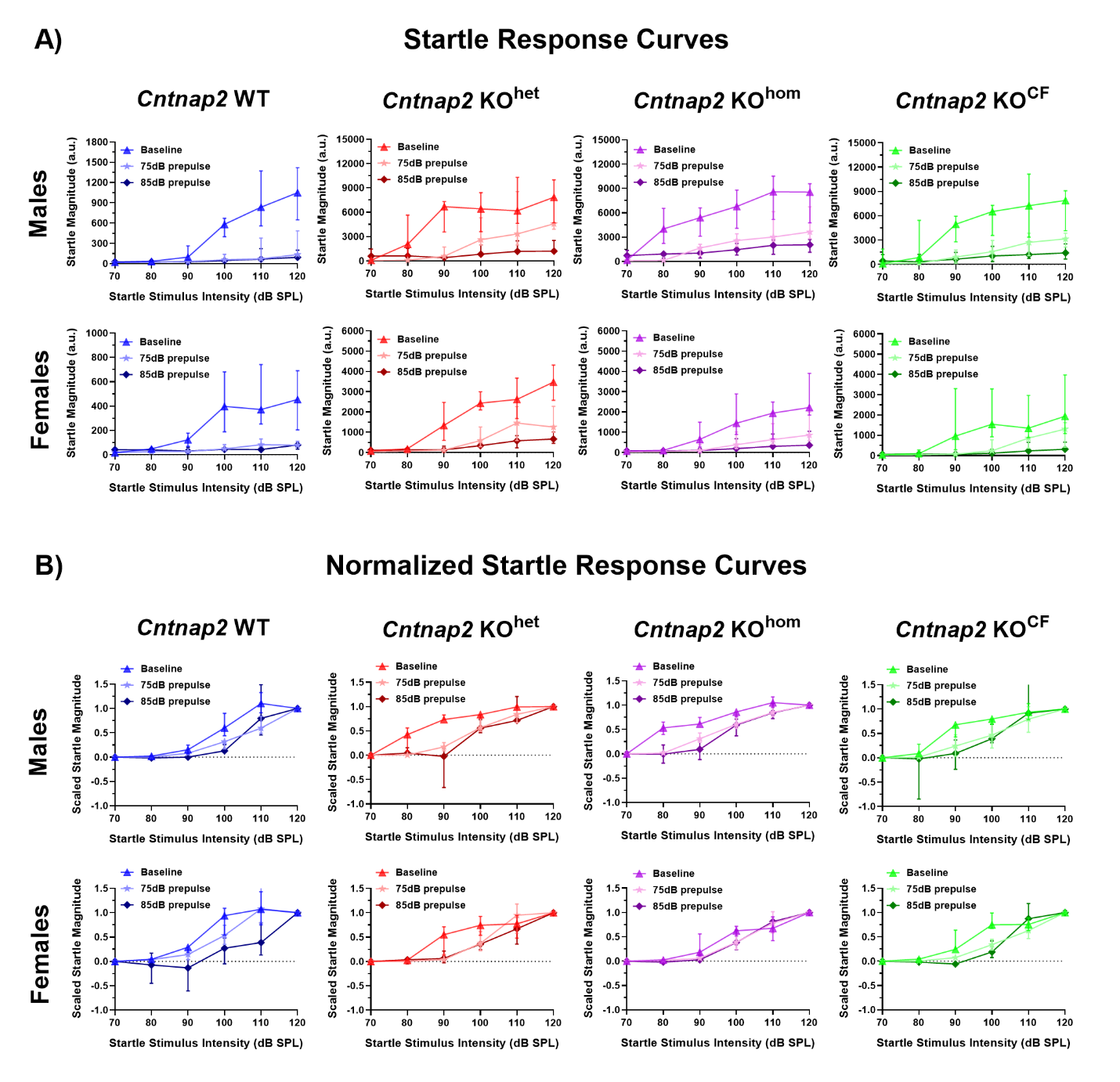


**Supplementary Figure 7.** PPI curves showing startle and sound scaling. **(A)** Startle response curves for baseline and prepulse conditions (75 dB and 85 dB). Note: Y-axis scales for *Cntnap2* WT and KO rats are different as KO rats have a greater baseline startle response magnitude than WT rats. Goodness of fit Sy.x: male WT baseline = 449.7, 75 dB = 249.6, 85 dB = 54.27; male KO^het^ baseline = 2485, 75 dB = 2192, 85 dB = 673.5; male KO^hom^ baseline = 2413, 75 dB = 1401, 85 dB = 681.3; male KO^CF^ baseline = 2736, 75 dB = 931.3, 85 dB = 870.3; female WT baseline = 181.1, 75 dB = 44.16, 85 dB = 27.03; female KO^het^ baseline = 841.6, 75 dB = 752.8, 85 dB = 223.3; female KO^hom^ baseline = 932.0, 75 dB = 570.6, 85 dB = 253.0; female KO^CF^ baseline = 2223, 75 dB = 487.0, 85 dB = 462.4. **(B)** Scaled startle response curves for baseline and prepulse conditions (75 dB and 85 dB). Goodness of fit Sy.x: male WT baseline = 0.2048, 75 dB = 0.09425, 85 dB = 0.2019; male KO^het^ baseline = 0.1263, 75 dB = 0.1606, 85 dB = 0.4919; male KO^hom^ baseline = 0.1311, 75 dB = 0.1013, 85 dB = 0.1928; male KO^CF^ baseline = 0.1673, 75 dB = 0.1783, 85 dB = 0.4585; female WT baseline = 0.1956, 75 dB = 0.2305, 85 dB = 0.9432; female KO^het^ baseline = 0.1961, 75 dB = 0.1977, 85 dB = 0.1843; female KO^hom^ baseline = 0.1823, 75 dB = 0.1925, 85 dB = 0.1223; female KO^CF^ baseline = 0.2003, 75 dB = 0.1090, 85 dB = 0.2101.


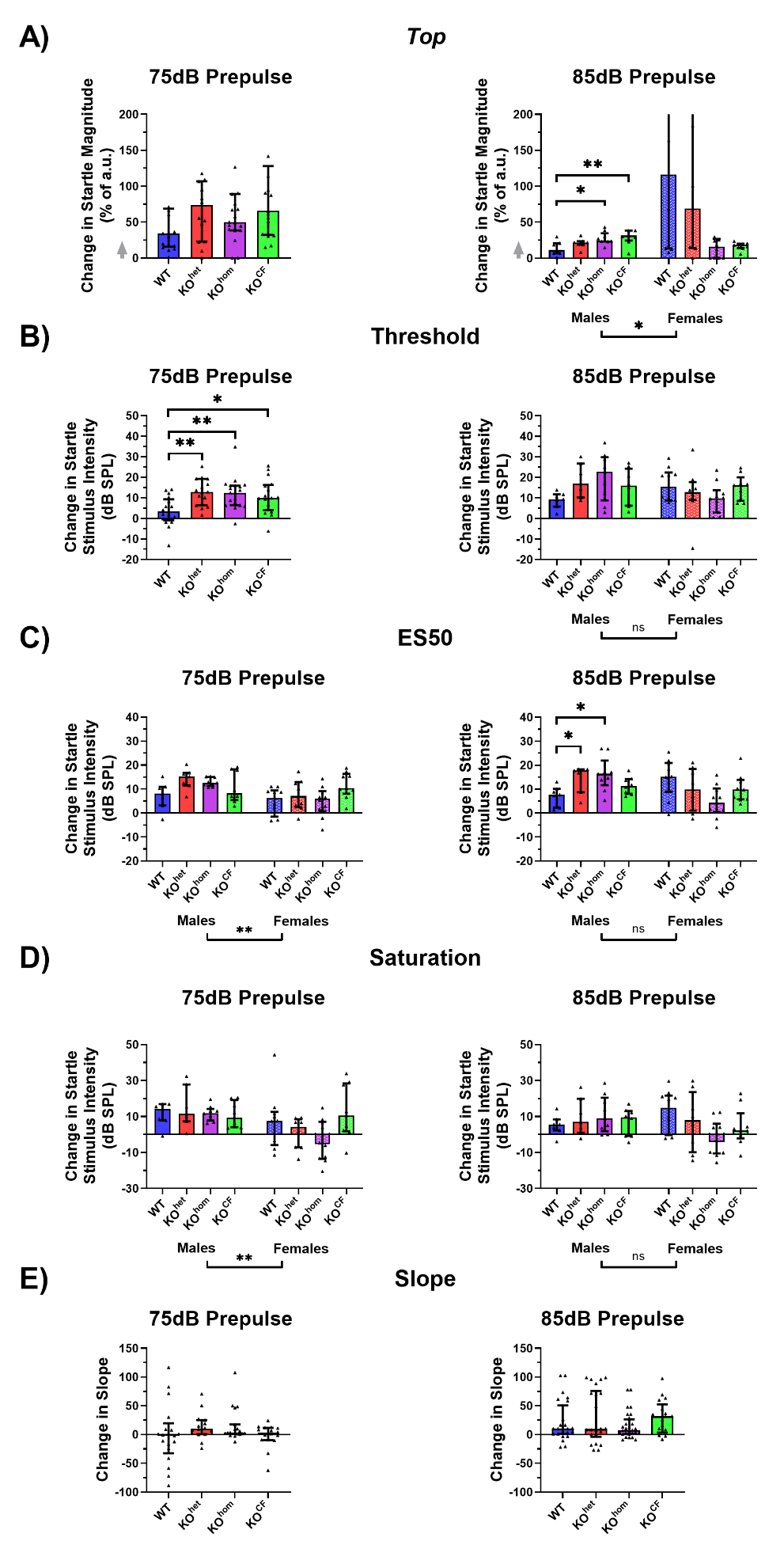


**Supplementary Figure 8.** PPI startle and sound scaling parameters. For measures where there was no effect of sex or interaction effects involving sex, data was collapsed across sex. **(A)** Maximum Startle Response (*Top*). The grey arrows indicate that there are values outside the limits of the y-axis, but graphs were zoomed in to visualize the data more clearly. With a 75 dB prepulse, there was no effect of genotype on the change in the *Top* (*p* = 0.2305, *F*(3, 64) = 1.472). With an 85 dB prepulse, there was an effect of genotype on the change in the *Top* for males (*p* = 0.0006, *F*(3, 27) = 5.105). *Cntnap2* KO^hom^ and KO^CF^ males had a greater change in *Top* than WT males (*Cntnap2* KO^hom^ *p* = 0.0225, *Cntnap2* KO^CF^ *p* = 0.0096). **(B)** Change in threshold with a prepulse from baseline. With a 75 dB prepulse, there was an effect of genotype on the change in the threshold for males (*p* = 0.0013, *F*(3, 64) = 5.917). All *Cntnap2* KO rats had a greater change in threshold than WT rats (*Cntnap2* KO^het^ *p* = 0.0026, *Cntnap2* KO^hom^ *p* = 0.0037, *Cntnap2* KO^CF^ *p* = 0.0388). With an 85 dB prepulse, there was no effect of genotype on the change in the threshold for males (*p* = 0.1140, *F*(3, 27) = 2.176) or females (*p* = 0.5130, *F*(3, 33) = 0.7810). **(C)** Change in ES50 with a prepulse from baseline. With a 75 dB prepulse, there was no effect of genotype on the change in the ES50 for males (*p* = 0.0810, *F*(3, 27) = 2.498) or females (*p* = 0.0707, *F*(3, 33) = 2.574). With an 85 dB prepulse, there was a significant effect of genotype on the change in ES50 for males (*p* = 0.0158, *F*(3, 27) = 4.120) and this effect was trending towards significance for females (*p* = 0.0669, *F*(3, 33) = 2.623). *Cntnap2* KO^het^ and KO^hom^ males, but not KO^CF^ males, had a greater change in ES50 than WT males (*Cntnap2* KO^het^ *p* = 0.0325, *Cntnap2* KO^hom^ *p* = 0.0228, *Cntnap2* KO^CF^ *p* = 0.4392). **(D)** Change in saturation with a prepulse from baseline. For both prepulses, there was no effect of genotype on the change in saturation for males (75 dB *p* = 0.9474, *F*(3, 27) = 0.1202, 85 dB *p* = 0.7313, *F*(3, 27) = 0.4327) or females (75 dB *p* = 0.0869, *F*(3, 33) = 2.385, 85 dB *p* = 0.1204, *F*(3, 33) = 2.090). **(E)** Change in slope with a prepulse from baseline. For both prepulses, there was no effect of genotype on the change in slope (75 dB *p* = 0.1257, *F*(3, 64) = 1.981, 85 dB *p* = 0.6418, *F*(3, 64) = 0.5624). *p < 0.05, **p < 0.01, ***p < 0.0001, ns indicates non-significance of the comparison.
